# Myeloid cell interferon secretion restricts Zika flavivirus infection of developing and malignant human neural progenitor cells

**DOI:** 10.1016/j.neuron.2022.09.002

**Published:** 2022-09-28

**Authors:** Harry Bulstrode, Gemma C Girdler, Tannia Gracia, Alexander Aivazidis, Ilias Moutsopoulos, Adam MH Young, John Hancock, Xiaoling He, Katherine Ridley, Zhaoyang Xu, John H Stockley, John Finlay, Clement Hallou, Teodoro Fajardo, Daniel M Fountain, Stijn van Dongen, Alexis Joannides, Robert Morris, Richard Mair, Colin Watts, Thomas Santarius, Stephen J Price, Peter JA Hutchinson, Emma J Hodson, Steven M Pollard, Irina Mohorianu, Roger A Barker, Trevor R Sweeney, Omer Bayraktar, Fanni Gergely, David H Rowitch

**Affiliations:** 1 Wellcome MRC Cambridge Stem Cell Institute; University of Cambridge, Cambridge, CB2 0AW, UK; 2 Division of Academic Neurosurgery, Department of Clinical Neurosciences; University of Cambridge, Cambridge, CB2 0QQ, UK; 3 Cancer Research UK Cambridge Institute; University of Cambridge, Cambridge, CB2 0RE, UK; 4 Wellcome Sanger Institute, Hinxton, CB10 1SA, UK; 5 Department of Paediatrics; University of Cambridge, Cambridge, CB2 0QQ, UK; 6 Department of Virology; University of Cambridge, Cambridge, CB2 0QQ, UK; 7 Department of Virology; Royal London Hospital, Barts Health NHS Trust, London, E1 2ES, UK; 8 Manchester Centre for Clinical Neurosciences, Manchester, M6 8HD, UK; 9 Institute of Cancer and Genomic Sciences, University of Birmingham, Birmingham, B15 2SY, UK; 10 Experimental Medicine and Immunotherapeutics; University of Cambridge, Cambridge, CB2 0QQ, UK; 11 Centre for Regenerative Medicine and Cancer Research UK Edinburgh Centre, Institute for Regeneration and Repair, University of Edinburgh, Edinburgh EH16 4UU, UK; 12 The Pirbright Institute, Guildford, Surrey, GU24 0NF, UK; 13 Department of Biochemistry; University of Oxford, Oxford, OX1 3QU, UK

**Keywords:** Glioblastoma, GBM, neural, Zika, flavivirus, oncolytic, microglia, macrophage, myeloid, interferon, cytokine

## Abstract

Zika Virus (ZIKV) can infect human developing brain (HDB) progenitors resulting in epidemic microcephaly, whereas analogous cellular tropism offers treatment potential for the adult brain cancer, glioblastoma (GBM). We compared productive ZIKV infection in HDB and GBM primary tissue explants that both contain SOX2+ neural progenitors. Strikingly, whereas HDB proved uniformly vulnerable to ZIKV infection, GBM was more refractory and this correlated with an innate immune expression signature. Indeed, GBM-derived CD11b+ microglia/macrophages were necessary and sufficient to protect progenitors against ZIKV infection in a non-cell autonomous manner. Using SOX2+ GBM cell lines, we found that CD11b+ conditioned medium containing type 1 interferon beta (IFNβ) promoted progenitor resistance to ZIKV, whereas inhibition of JAK1/2 signaling restored productive infection. Additionally, CD11b+ conditioned medium and IFNβ treatment rendered HDB progenitor lines and explants refractory to ZIKV. These findings provide insight into neuroprotection for HDB progenitors as well as enhanced GBM oncolytic therapies.

## Introduction

Viral infections of human brain during early development can result in congenital pathology, death and lifelong disability (Megli and Coyne, 2021). A recently recognized congenital (aka, ‘TORCH’) infection is caused by Zika virus (ZIKV), a mosquito-borne positive strand RNA flavivirus. An estimated five to ten percent of babies born to mothers infected during pregnancy with ZIKV epidemic strains exhibit features of Zika Congenital Syndrome including microcephaly and/or neurodevelopmental delay (Cugola et al., 2016), whereas few sequelae of ZIKV infection have been reported in the adult brain (Carod-Artal, 2018).

ZIKV epidemic strains including PE243 (ZIKV-PE243), identified in Brazil in 2015 and the basis for our studies (Donald et al., 2016), have been shown to infect human neural progenitor cells marked by expression of SOX2 (Chavali et al., 2017; Li et al., 2016; Retallack et al., 2016), an SRY-box transcription factor (Wegner and Stolt, 2005). Alongside their developmental role, SOX2 and related neural lineage transcription factors drive proliferation and invasion of cancer stem cells in malignant primary brain tumours including glioblastoma (GBM) (Bulstrode et al., 2017; Sturm et al., 2014; Suvà et al., 2014). Glioma stem cells are resistant to host immune attack and to current treatment (Bao et al., 2006; Gangoso et al., 2021; Mehta et al., 2011), suggesting them as a prime target for novel GBM therapy (Gimple et al., 2019; Suvà and Tirosh, 2020; Wang et al., 2020). In this regard, repurposing pathogenic viruses offers treatment promise, directly through cancer stem cell lysis and indirectly through immune stimulation (Harrington et al., 2019). ZIKV has demonstrated oncolytic potential in GBM, prolonging survival in a mouse GBM model (Zhu et al., 2017). However, virus-based strategies have yet to demonstrate consistent clinical benefit in brain cancer (Zhang and Liu, 2020), in part reflecting limitations in the available cell and animal models, especially in capturing the human tissue microenvironment (Liu et al., 2013).

SOX2 expression has been reported to mark diverse ZIKV target cells including neural progenitor cells, oligodendrocyte precursor cells, astrocytes (Garcez et al., 2016; Retallack et al., 2016; Souza et al., 2016), and glioma stem cells(Chen et al., 2018; Zhu et al., 2017, 2020). Nevertheless, human embryonic stem cells and iPSCs are not highly permissive to ZIKV infection, despite high SOX2 expression and proliferative capacity (Tang et al., 2016), suggesting additional factors are involved. Here, we investigated productive ZIKV infection in progenitor cell populations in primary HDB versus GBM. We found striking differences in relative rates of infection between these explants that could not simply be accounted for by cell-intrisic factors such as SOX2 expression levels. We go on to show that non-cell-autonomous microenvironmental factors secreted from myeloid cells are both necessary and sufficient to regulate productive infection of HDB and GBM progenitor cells.

## Results

### SOX2+ progenitor cells in human developing brain are vulnerable to ZIKV infection whereas GBM progenitors are refractory

**Figure 1:**
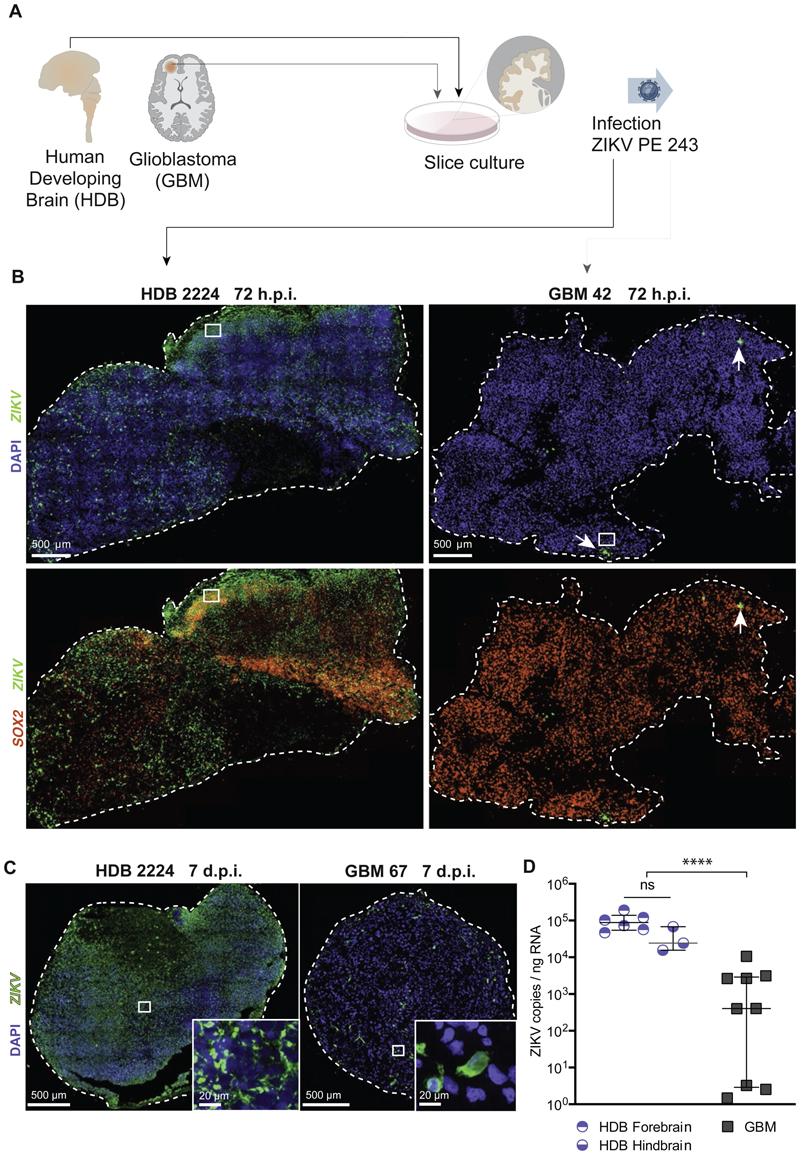
Human developing brain organotypic slices are vulnerable to ZIKV whereas GBM slices are refractory. **(A)** Primary HDB and GBM samples were processed for slice culture, then infected with ZIKV PE243. **(B)** RNAscope smFISH of representative ZIKV-infected HDB and GBM slice cultures with ZIKV 1x10^7 PFU, 72 hours post infection (h.p.i.), using SOX2 (red) and ZIKV (green) probes. White arrow denote ZIKV infection. **(C)** smFISH of ZIKV-infected HDB and GBM slice cultures with ZIKV 1x10^7 PFU, 7 days post infection (d.p.i.), using ZIKV probe (green). **(D)** RT-qPCR of ZIKV in HDB and GBM slice cultures, infected with ZIKV 1x10^7 PFU, 7 d.p.i., (Median and interquartile range indicated. **** Mann Whitney U t-test p <0.0001). Data points represent replicate slices from HDB *n=*2 specimens, both with hb and fb regions, and GBM *n=*3 patients.

We first compared rates ZIKV-PE243 productive infection in explant slice cultures from HDB (9-12-weeks gestational age, *n=*2 specimens, including forebrain and hindbrain regions from both specimens) and human adult GBM (*n=*3 patient samples). As shown (Figure 1A, B; Suppl. Figure S1A-D), while HDB samples were permissive to infection, GBM slices were refractory to ZIKV, as measured by single molecule fluorescent *in situ* hybridization (smFISH) and immunofluorescence (IF) at 72-hours post-infection (h.p.i.). We noted that infection tended to be patchy and restricted to cells at the tissue surface at 48-72-h.p.i. in both HDB (Figure S1A) and GBM. In view of the longer time needed for ZIKV to penetrate thick tissues slices in culture and to be consistent with prior study endpoints (Zhu et al., 2017; Muffat et al., 2018), we also scored 7-days post-infection (7 d.p.i.; Figures 1C, D; S1A, E, F). Quantitative polymerase chain reaction (RT-qPCR) assay of viral genome copy number confirmed a significant difference in productive virus levels between HDB and GBM samples at 7 d.p.i (Figure 1D), at which point HDB explant tissue showed more extensive ZIKV infection and apoptosis compared to GBM tissue that was relatively intact (Figure S1E,F).

To assess the potential of cell-intrinsic contributions to differential ZIKV vulnerability in progenitor cells, we quantified virus levels by RT-qPCR 48 hours post infection adherent serum-free cultures of HDB cell lines derived from forebrain and hindbrain at 10-(HDB FB1 and HB1) or 12-weeks (HDB FB12 and HB12) gestation, as well as cell lines from paediatric diffuse H3K27M pontine midline glioma (DMG 007, DMG B117, DMG B169), and cell lines derived from adult GBM (GBM E22, GBM E25, GBM E34). In keeping with previous reports using cell lines (Zhu et al., 2017, 2020), productive viral infection and consequent lytic cell death ensued in GBM as well as in HDB cell lines over 48-96 hours (Figure S2A, B). The cell lines began to detach at 48-72 h.p.i. compromising the accuracy of RT-qPCR assays beyond these timepoints. The finding that GBM progenitor lines were vulnerable to ZIKV – in contrast to findings with refractory primary GBM slice explants – initially suggested a role for non-cell autonomous regulation by microenvironmental factors.

**Figure 2:**
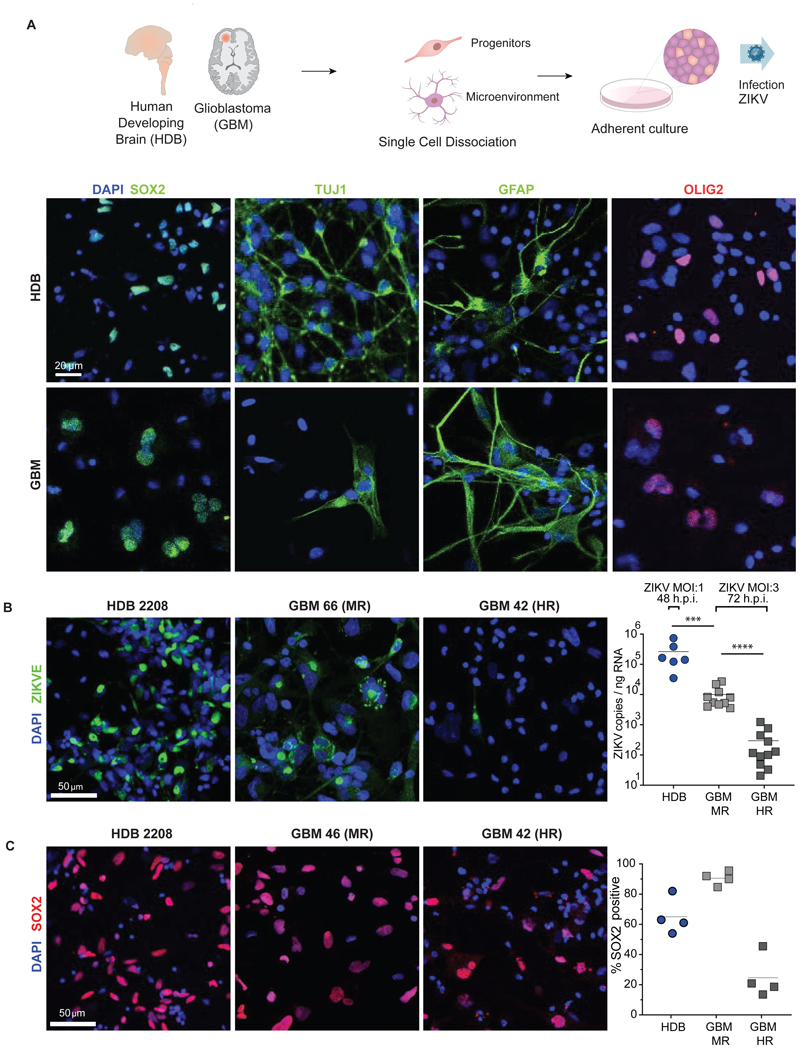
SOX2+ target cell composition alone cannot account for HDB/GBM ZIKV infection/resistance **(A)** Primary HDB and GBM tissue was subject to enzymatic dissociation followed by plating in serum-free adherent culture. Representative IF of key lineage markers in primary HDB and GBM dissociated adherent cultures is shown (see also Figure S2C). **(B)** Representative IF (left) and RT-qPCR (right) of 6 HDB and 20 GBM dissociated adherent cultures. Highly refractory and moderately refractory GBM cultures were defined with reference to the median ZIKV copy number for all GBMs analysed (ZIKV MOI:1 48 h and ZIKV MOI:3 72 h respectively). Each RT-qPCR data point represents the average copy number across three replicate wells/slices per sample. (Mann-Whitney test *** = p <0.001, **** = p<0.0001) **(C)** Representative IF (left) and % cells SOX2+ in HDB (*n=*4 specimens), HR GBM (*n=*4 patients) and MR GBM (*n=*4 patients) adherent cultures (left) and. Cell counts for IF and smFISH determined by manual counting of at least 200 cells per condition. Mean values across all biological replicates for each condition are indicated by horizontal lines. (ANOVA p< 0.0001)

We next dissociated primary HDB and GBM tissue to single cell suspension, generating adherent primary cultures comprising diverse cell identities (Figure 2A; Figures S2C-E). We observed that all six HDB cultures tested (8-9 weeks gestational age, including one sample pair from fore- and hindbrain of a single specimen) showed productive ZIKV infection by RT-qPCR (Figure 2B). We performed similar ZIKV infection on 20 primary dissociated GBM cultures and found that productive viral infection was 10-100 fold lower than HDB. This necessitated threefold higher multiplicity of infection (MOI 3) in GBM versus HDB (MOI1) and longer exposure in GBM (72 h) versus HDB (48 h) for GBM to achieve detectable levels of productive infection. Indeed, relative to HDB, all GBM cultures were refractory to ZIKV infection. Moreover, we could clearly divide GBMs into moderately refractory (MR) or highly refractory (HR) to infection based on viral RNA levels by RT-qPCR (Figure 2B).

Consistent with prior findings of SOX2 being a key determinant of ZIKV infection (Zhu et al., 2020), the proportion of cells positive for SOX2 was higher in MR than HR GBM samples (Figure 2C; Figure S2E), whereas there was no significant difference in the fraction of cells labelled by the proliferation marker MKI67 (Figure S2F). Interestingly, despite higher productive infection in HDB samples, the proportion of SOX2+ cells was higher in MR GBM than in HDB (Figure 2C), indicating that the SOX2+ composition alone could not account for differential vulnerability in HDB versus GBM. Together, these findings suggested that non-SOX2 cell autonomous (e.g., microenvironment) factors regulated ZIKV progenitor cell infection.

### ZIKV-refractory GBM displays an innate immune signature

**Figure 3:**
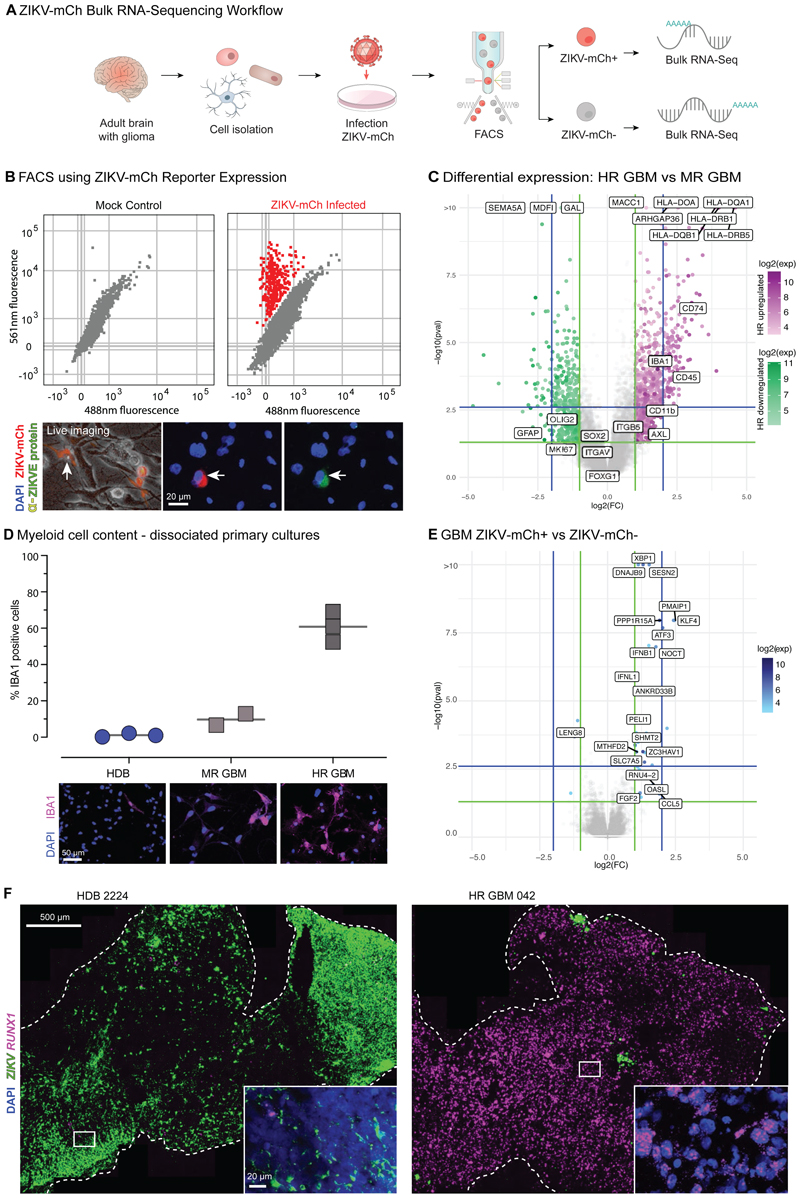
RNA-Sequencing identifies an innate immune signature in ZIKV-refractory GBM. **(A)** Experimental scheme: dissociated primary HDB and GBM cultures were infected with ZIKV-mCh reporter virus, then sorted by FACS into mCherry negative and positive fractions for sequencing as shown. **(B)** ZIKV-mCherry reporter (ZIKV-mCh) coincides with flavivirus envelope protein expression (α-ZIKVE protein Sigma MAB10216), marking the ZIKV infected fraction. **(C)** edgeR differential expression analysis of bulk RNA-Seq in HR GBM (*n=*3 Cherry paired libraries) vs MR GBM (*n=*4 Cherry positive/negative paired libraries). Key glioma cell and myeloid cell signature genes are highlighted. HLA-Class II antigen-presenting genes and myeloid cell markers are highlighted. **(D)** Cell counts and representative IF images for myeloid cell marker IBA1 in dissociated cell cultures (n=3 each). **(E)** edgeR differential expression ZIKV-mCh+ vs ZIKV-mCh- primary GBM culture fractions. **(F)** ZIKV-infected HDB and GBM slice cultures: smFISH for ZIKV and myeloid cell marker RUNX1.

We next infected freshly dissociated primary HDB and GBM cultures with a ZIKV-mCherry (ZIKV-mCh) live transgenic reporter virus (Mutso et al., 2017), then performed fluorescence activated cell sorting (FACS) and bulk RNA-sequencing of both ZIKV-mCh-positive (ZIKV-mCh+) and -negative fractions (Figures 3A and B; Figure S3A). As expected, MR GBMs yielded higher ZIKV-mCh+ fractions than HR GBMs (Figure S3B).

We next used expression data to ask whether cell-intrinsic factors beyond SOX2 expression could account for refractory HR versus MR GBM findings. The Cancer Genome Atlas (TCGA) uses expression profiling to ascribe ‘proneural’, ‘classical’ and ‘mesenchymal’ sub-type identities across an extensive panel of bulk GBM tissue samples (Brennan et al., 2013). However, as shown (Figure S3B, C) these subtypes were not predictive of infection. Expression of neural stem cell markers SOX2 and MSI-1, as well as surface receptors Integrin αvβ5 and AXL, is associated with ZIKV permissivity (Chavali et al., 2017; Chen et al., 2018a; Garcez et al., 2016; Retallack et al., 2016; Souza et al., 2016; Zhu et al., 2017, 2020). These genes were highly expressed in the tumours analysed but we found no correlation between the level of bulk tissue expression and infection levels (Figure 3C).

While such cell-intrinsic expression features of GBM progenitors did not account for rates of ZIKV infection, we observed that HR GBM segregated both from MR GBM and HDB based on innate immune system signature genes such as *human leucocyte antigen* (*HLA*), *CD74, IBA1, CD45* and *CD11b*, a marker of myeloid lineage (Figure 3C; Figure S3D). Indeed, deconvolution analysis identified a glioma myeloid cell signature, which was strongest in HR GBM (Figure S3E). We used IF and smFISH to confirm that IBA1+ and *RUNX1*+ myeloid cell populations were increased in the HR GBM slices and dissociated cultures compared to MR GBM; indeed, myeloid cells were nearly undetectable in HDB (Figure 3D; Figure S3 D-F). Across the range of primary cultures assayed, bulk ZIKV-mCh+ fractions were enriched for expression of pro-inflammatory cytokines *IFNB1, IFNL1* and *CCL5* when compared to ZIKV-mCh-fractions (Figure 3E). To validate these findings, we analysed ZIKV infection and myeloid cell marker expression in primary slice cultures. *RUNX1* expression was almost entirely absent in ZIKV-high HDB slices, whereas it was strongly enriched in HR GBM slices (Figure 3F). Some myeloid cell populations are vulnerable to ZIKV infection (Meertens et al., 2017; Quicke et al., 2016; Wang et al., 2018; Xu et al., 2020), but there is also evidence that monocyte-derived macrophages at least can effectively restrict intrinsic Zika replication (Yang et al., 2021). Therefore the close correlation observed between tissue myeloid cell content and ZIKV refractory behaviour in our models invited closer analysis of the interactions of glioma cells with myeloid cells and their effects on infection dynamics.

**Figure 4:**
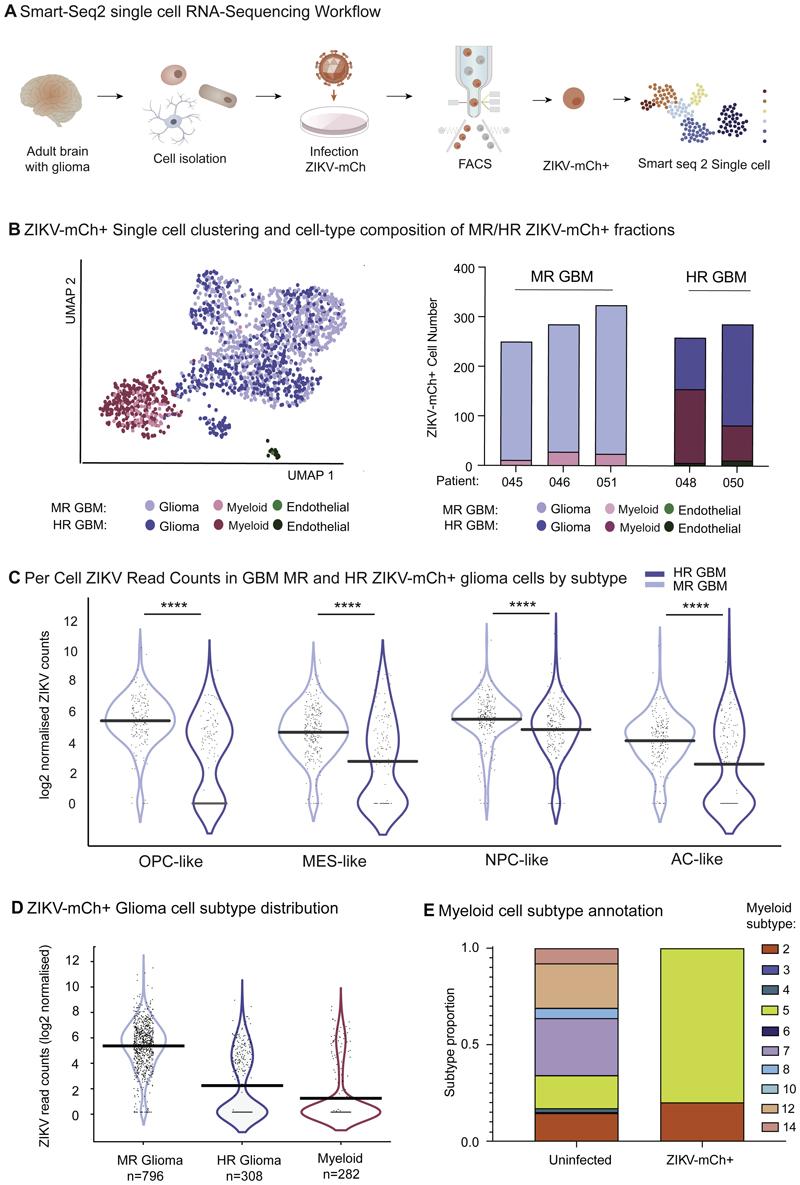
ZIKV productively infects diverse glioma stem cell identities. Infection is modulated by TME myeloid cells, and these adopt pro-inflammatory anti-viral states in response to ZIKV-mCh+ uptake. **(A)** Plate-based Smart-Seq2 single cell analysis of FACS sorted ZIKV-mCh+ GBM cells demonstrates predominance of glioma cell and myeloid cell identities. **(B)** Proportions of each cell type captured in 3 MR GBM and 2 HR GBM ZIKV-mCh+ single cell libraries. **(C)** Per cell normalised ZIKV read counts by glioma cell subtype: oligodendrocyte precursor cell-like (OPC-like), mesenchymal-like (MES-like), neural precursor cell-like (NPC-like), astrocyte-like (AC-like) (Neftel et al., 2019) and by parent tumour ZIKV resistance for all ZIKV-mCh+ glioma cells captured (horizontal bar indicates mean normalised transcript count per cell). Wilcoxon rank test p< 0.00001 for all cell subtype GBM MR vs HR comparisons. **(D)** Summary per cell normalised ZIKV read counts for ZIKV-mCh+ glioma cells in MR and HR GBM, and for myeloid cells from all tumours pooled (horizontal bar indicates mean normalised transcript count per cell; Wilcoxon signed rank test glioma cell (HR GBM) vs glioma cell (MR GBM) p = 8.3e-64). **(E)** Myeloid cell transcriptional subtypes comprising uninfected parent tumour samples (profiled by 10x RNA-Seq) and ZIKV-mCh+ myeloid cells (Smart-Seq2). Clusters according to (Sankowski et al., 2019): clusters 2 and 5 correspond to pro-inflammatory anti-viral states.

To this end, we performed single cell RNA sequencing (scRNA seq) of ZIKV-mCh+ FACS-purified cells from six primary GBM samples (Figure 4A). These data showed expression of the virus reporter across the range of glioma cell subtypes and in myeloid cells, the sole ZIKV-mCh+ immune cell population identified (Figure S4A, B and C). The proportion of myeloid cells was over-represented in HR GBM samples versus MR GBM (Figure 4B), and we noted that proneural GBM cell identities (OPC-like and NPC-like) distinguished MR GBM compared to HR GBM libraries (Figure S4D; χ^2^ = 34.1 p< 0.0001) (Neftel et al., 2019). We next amalgamated scRNA seq data from each patient cell fraction into ‘pseudobulk’ RNA-Seq libraries (Squair et al., 2021), allowing comparison of ZIKV levels using a DE-Seq2 model incorporating cell autonomous (glioma cell subtype) and microenvironment (MR GBM vs HR GBM) determinants. Despite the apparent trend to higher virus levels in proneural (oligodendrocyte precursor cell-like and neural progenitor cell-like) glioma cell subtypes (Figure 4C), overall no significant association between glioma cell subtype and ZIKV read count was demonstrated (DE-Seq2, p>0.05 all pairwise comparisons). On the other hand, cell fractions from MR GBM tumours demonstrated significantly higher ZIKV read counts compared to HR GBMs (log2fc 2.51, p<0.0001). Furthermore, ZIKV read counts were significantly higher in SOX2-expressing ZIKV-mCh+ cells than in those without SOX2 reads (Figure S4E). ZIKV read counts were lowest of all in ZIKV-mCh+ myeloid cells themselves (Figure 4D). The latter is consistent with recent findings that myeloid cells can internalize oncolytic herpes simplex virus and express reporter genes without supporting viral replication (Delwar et al., 2018). Whereas snap-frozen uninfected GBM controls demonstrated a broad range of myeloid cell cell identity states (Sankowski et al., 2019), ZIKV-mCh+ myeloid cells exhibited exclusively pro-inflammatory anti-viral states (Figure 4E). Taken together, we conclude that variation in ZIKV levels between HR and MR GBM-derived glioma cells (Figure 4D) greatly exceeds that associated with proneural vs non-proneural cell identity (Figure 4C), again suggesting that tumour microenvironment (TME) rather than cell-intrinsic factors could be the key determinant of ZIKV progenitor cell infection.

### Tumour myeloid cells render GBM refractory to ZIKV infection

**Figure 5:**
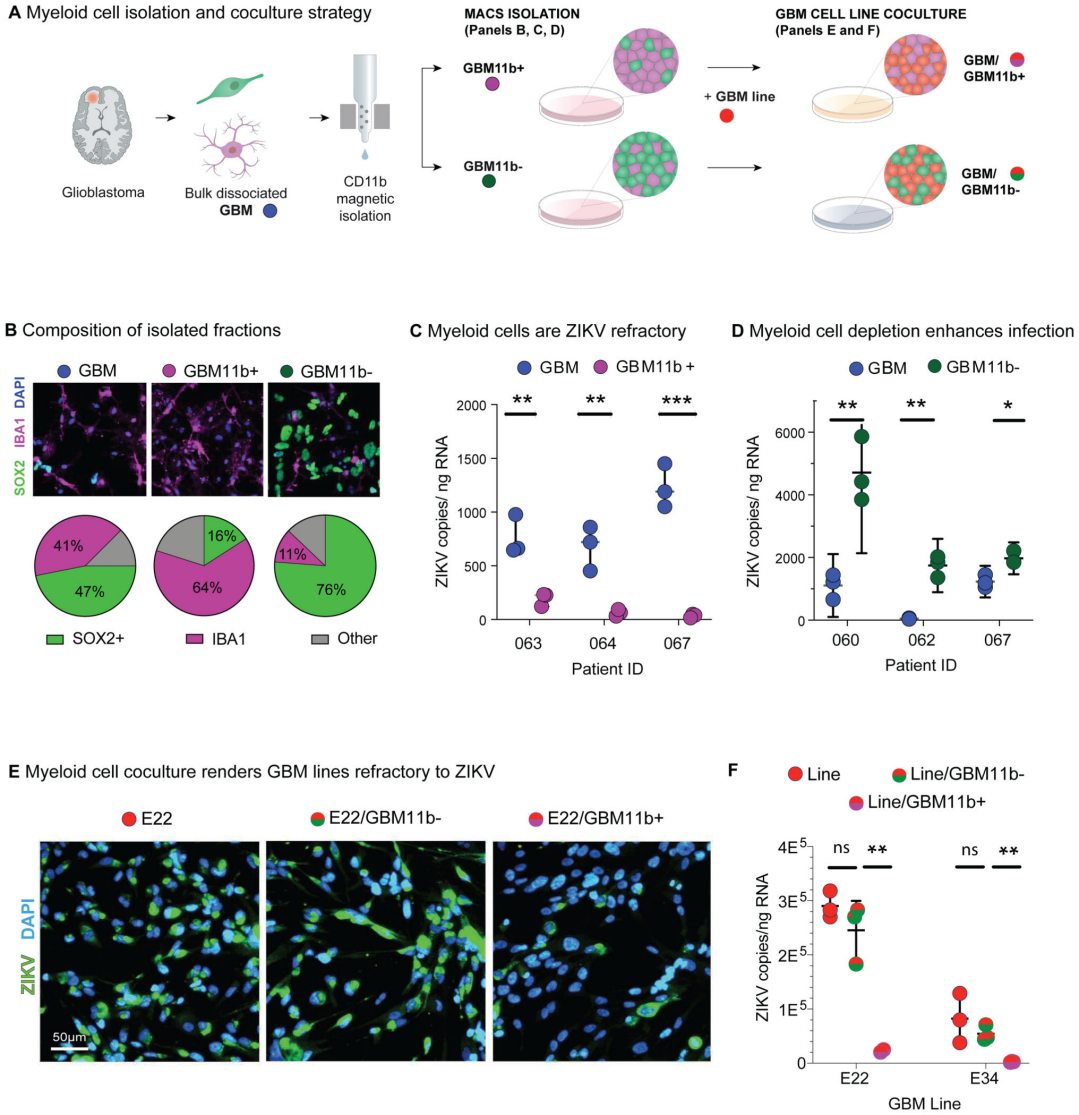
Glioma myeloid cells confer ZIKV resistance. **(A)** Primary GBM samples were dissociated to single-cell suspension and plated in bulk (GBM), or first separated into CD11b-enriched (GBM11b+) and CD11b-deplete fractions (GBM11b-) using antibody-conjugated magnetic bead and column-based sorting. These fractions were assayed directly, or in coculture with HDB and GBM lines. **(B)** Representative IF images of SOX2+ glioma cells and IBA1+ myeloid cells in GBM, GBM11b+ and GBM11b-fractions, and average proportion of each cell type across *n=*4 matched bulk and sorted GBM fractions. **(C)** ZIKV PE243 copy number assayed by RT-qPCR in paired infected GBM and GBM11b+ primary cultures (*n=*3). **(D)** ZIKV PE243 copy number assayed by RT-qPCR in paired infected GBM and GBM11b-primary cultures (*n=*3). **(E)** GBM E22 line infected in isolation, or in coculture with GBM11b- or GBM11b+ fractions at a 2:1 ratio, then fixed at 48 h.p.i. for IF (See also Figure S5). **(F)** GBM E22 line infected in isolation, or in coculture with GBM11b- or GBM11b+ fractions, processed for RNA extraction and RT-qPCR viral copy number assay (unpaired t-test *p<0.05; ** p<0.01; ***p<0.001) (See also Figure S5).

Findings above prompted us to directly test function of the myeloid cells over-represented in the HR GBM tumor microenvironment (TME). We purified TME myeloid cells from primary GBM cultures (*n*=7 independent samples) using CD11b magnetic activated cell sorting (MACS) (Figure 5A), which yielded live CD11b-positive GBM fractions (GBM11b+) enriched for a variety of IBA1+ myeloid cells (Figure S4F), and depleted for SOX2+ glioma cells, as well as CD11b-depleted GBM fractions (GBM11b-) exhibiting a higher SOX2/IBA1 ratio (Figure 5B; Figure S5A) compared to parental GBM cultures.

We first analysed productive ZIKV infection of the GBM11b+ fraction (i.e., TME myeloid cells alone) compared with the bulk parental GBM cells. As shown (Figure 5C), we found that RNA copies were significantly reduced in GBM11b+ fractions, indicating that myeloid cells were refractory to productive infection. Interestingly, GBM11b- fractions showed higher infection rates versus parental bulk GBM (Figure 5D), indicating that myeloid cell depletion enhanced productive infection. Thirdly, we tested the ability of myeloid cells to drive the permissive GBM cell lines (E22, E34) to a ZIKV-refractory state (Figure 1F; Figure S2A); we found that addition of GBM11b+ cells to GBM lines in a 1:2 ratio was associated with >ten-fold reduction in ZIKV levels (Figure 5E and F; Figure S5B). Finally, TME myeloid cells also rendered primary HDB cultures ZIKV-refractory (Figure S5C), suggesting a neuroprotective effect.

### Myeloid cell-secreted cytokines prevent progenitor cell ZIKV infection

**Figure 6:**
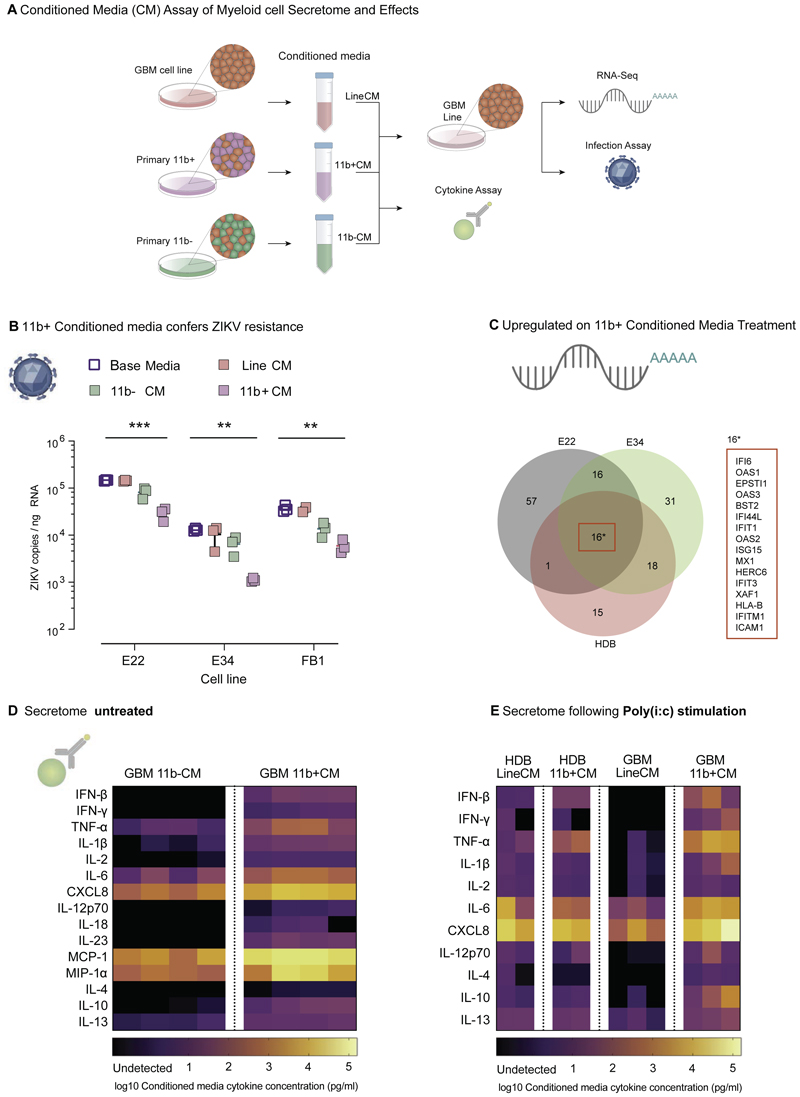
Glioma myeloid cells drive Zika resistance through cytokine secretion. **(A)** Conditioned media harvested from GBM11b+ fractions (11b+CM), from GBM11b-fractions (11b-CM) and from GBM lines (LineCM), profiled using Meso Scale Discovery U-Plex and V-Plex (MSD) cytokine panels, and applied to GBM lines for conditioned media functional assays and expression profiling. **(B)** ZIKV RT-qPCR 48 hours post infection (MOI = 1) of GBM lines GBM E22 and GBM E34 and HDB FB1 cells cultured in base media, LineCM, 11b-CM or 11b+CM. (2-way ANOVA cell line conribtion p < 0.0001 and conditioned media contribution p < 0.0001; Welch’s t-test Base media vs 11b+CM *** p<0.001, ** p<0.01) **(C)** Venn diagram summarises pattern of gene upregulation in GBM E22, GBM E34, and primary HDB cells (specimen BRC 2251) post-treatment with 11b+CM versus control LineCM. **(D)** MSD assay of cytokine content in 11b-CM and 11b+CM at 48 h in culture (n=4 paired GBM sample fractions from 4 patient tumours). **(E)** MSD assay of Pi:c-LineCM and Pi:c-11b+CM derived from the HDB and GBM fractions indicated, following stimulation with poly(I:C) viral dsRNA mimetic at 10 μg/ml.

We next investigated the molecular basis for non-cell autonomous myeloid cell-driven inhibition of glioma cell infection. To assay whether this mechanism involved secreted factors, we harvested filtered conditioned medium from the CD11b- and CD11b+ fractions of three primary GBM dissociated cell cultures, and from GBM E22 and GBM E34 lines (11b-CM, 11b+CM, LineCM respectively; Figure 6A). Small volumes of conditioned media were also obtained from sorted HDB CD11b+ fractions for cytokine assay (Figure S5D). Application of 11b+CM to GBM E22, GBM E34 and HDB-FB1 cell lines resulted in an approximately ten-fold reduction in ZIKV levels compared to control LineCM treatment (Figure 6B).

We used RNA sequencing to identify transcriptional changes in lines GBM E22, GBM E34 and primary HDB cells on treatment with 11b+CM. A total of 154 differentially expressed genes across these libraries were strongly enriched for interferon-stimulated genes (ISGs) by gene ontology analysis (Figure S5E). Furthermore, all 16 genes differentially expressed in the three cell lines were ISGs (Figure 6C). In contrast, expression of intrinsic ‘stemness’ genes such as *SOX2, OLIG2* and *FOXG1* in glioma cells was unaffected by 11b+CM treatment (Figure S5F), giving no reason to suspect that interferon treatment at these levels compromises self-renewal or viability.

Analysis of 11b+CM samples by Meso Scale Discovery (MSD) assay indicated significant enrichment for 15 cytokines compared to paired 11b-CM samples (*n=*4 each; paired t-tests) (Figure 6D). High levels of CXCL8, TNF-α, and IL-6, pro-inflammatory cytokines associated with intracellular pathogen killing and neutrophil recruitment (Dixit and Simon, 2012; Mantovani et al., 2004), would be predicted to contribute to anti-viral properties *in vivo*. The most striking fold change enrichment in 11b+CM samples, however, was for anti-viral cytokines including IFNβ. We observed further enrichment for these factors in conditioned media harvested from (i) GBM11b+ and (ii) HDB11b+ fractions pretreated with poly(I:C) to mimic the presence of viral RNA, denoting this poly(I:C) responsive conditioned media Pi:c_11b+CM (Figure 6E). Importantly, neither HDB nor GBM lines themselves were able to generate a comparable magnitude of secreted IFNβ response compared to myeloid cells (Figure 6E).

**Figure 7:**
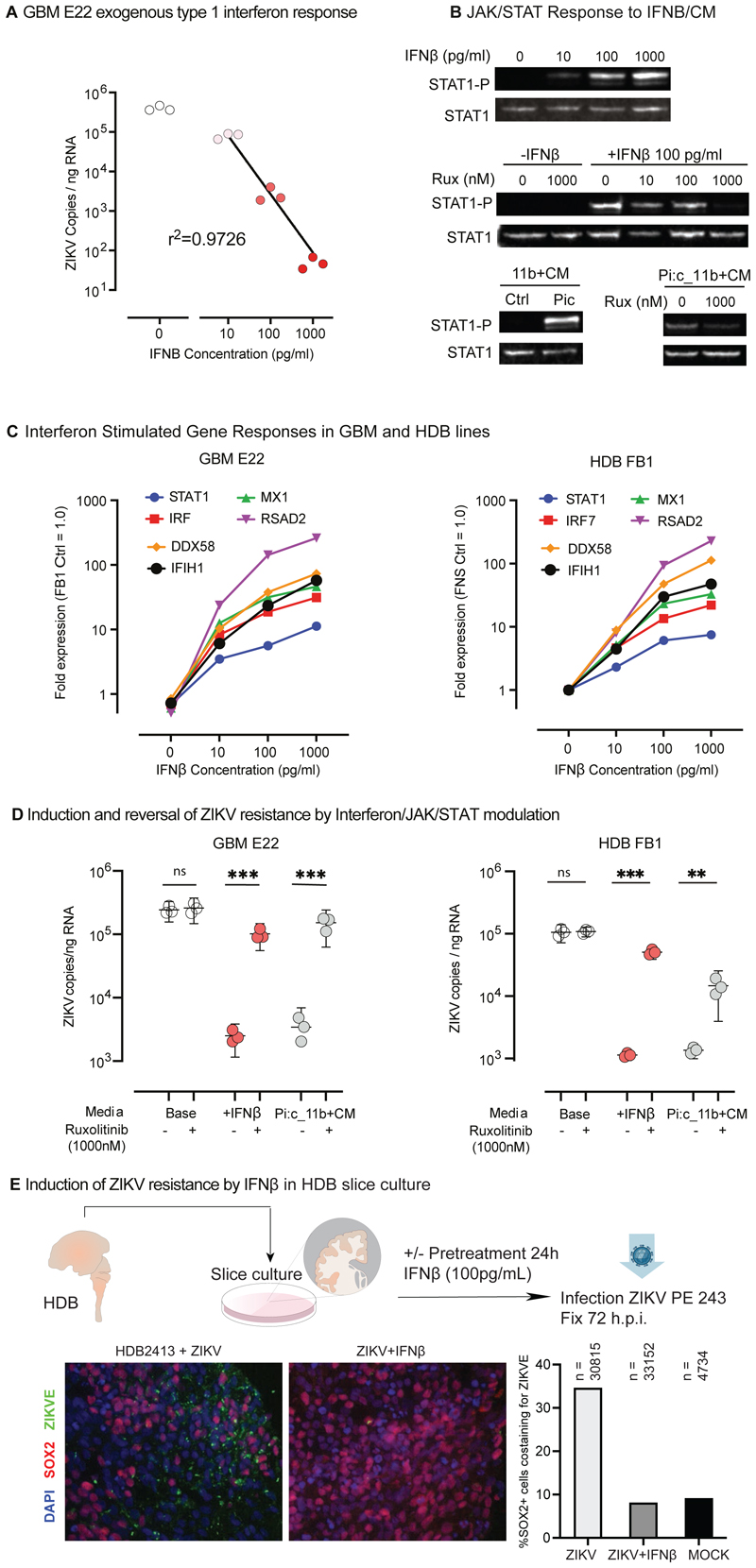
Myeloid cell secretome-induced resistance reflects IFNβ content and can be reversed by JAK/STAT inhibition **(A)** ZIKV RT-qPCR of GBM E22 cultures 48 h.p.i. treated with exogenous recombinant IFNβ as indicated. **(B)** STAT1 phosphorylation in GBM E22 cells harvested 3 hours post treatment with: *(Upper)* Recombinant IFNβ; *(Middle)* IFNβ +/-JAK1/2 inhibitor ruxolitinib; *(Lower)* 11b+CM and Pi:c-11b+CM +/-ruxolitinib (Rux). **(C)** Normalised expression by qRT-PCR for the interferon stimulated genes indicated in GBM E22 and HDB FB1 cultures, in response to exogenous IFNβ at the indicated media concentrations, normalised to GAPDH expression and with gene expression in untreated HDB FB1 cells assigned the value 1.0. **(D)** ZIKV RT-qPCR in HDB FB1 (left) and GBM E22 glioma stem cells (right) 48 h.p.i. or 48 hours with or without 24 hours pre-treatment with IFNβ (100 pg/ml) or Pi:c-11b+CM, +/-ruxolitinib 1000 nM. (unpaired t-test *p < 0.05, **p<0.01, ***p<0.001) **(E)** HDB slice cultures harvested at 72 h.p.i., infected with ZIKV +/-IFNβ. Graph shows the percentage of SOX2+ cells infected with ZIKV versus total SOX2+ cells for the conditions indicated, MOCK is uninfected. n = total cells analysed across 3 slices per condition, 1-3 replicate cryosections per slice, in 1 HDB sample.

We extended analysis of ZIKV neuroprotective effects to HBD progenitors (Figure 7) and observed robust dose-dependent ability of recombinant IFNβ to inhibit ZIKV production at concentrations equivalent to those found in 11b+CM harvested with or without poly(I:C) stimulation (Figure 7A), whereas treatment with control recombinant cytokines also present at high levels in our conditioned media was associated with no effect (Figure S5G). IFNβ drives JAK/STAT signalling and we found that addition of IFNβ or poly(I:C) 11b+CM drove dose-dependent phosphorylation of STAT1 in GBM cell lines (Figure 7B). In both lines, STAT1 phosphorylation was inhibited by the JAK1/2 inhibitor, ruxolitinib. Blunted or absent type 1 interferon responses have previously been reported in both pluripotent and tissue stem cell populations (Burke et al., 1978; Wu et al., 2018), so we next tested the ability of HDB cells and GBM lines to respond to IFNβ. In each case a robust dose-dependent interferon stimulated gene response was evident (Figure 7C). Furthermore pre-treatment of GBM E22 cells with IFNβ for 24 hours prior to ZIKV exposure abolished infection as assessed by IF, with infection rates restored by addition of ruxolitinib (Figure S5H). Likewise, RT-qPCR confirmed near total inhibition of productive ZIKV infection in both HDB and GBM lines on pre-treatment with Pi:c_11b+CM or IFNβ, and conversely infection was partially or completely restored by addition of ruxolitinib under these conditions (Figure 7D). Finally, we tested primary HDB explants to investigate the neuroprotective effects of IFNβ against ZIKV. As shown (Figure 7E), IFNβ treatment reduced infection of SOX2+ progenitors.

## Discussion

We cross-compared ZIKV infection rates in human primary samples of HDB versus patient GBM to better understand the determinants of neurotropic infection in normal and malignant progenitor populations. Our findings reveal a common regulatory role for the human myeloid secretome, and a potent neuroprotective effect of IFNβ against ZIKV for HDB progenitors cells. This work has special relevance given the history of ZIKV epidemic microcephaly in low- and middle-income countries, and the possibility of new epidemic ZIKV variants (Regla-Nava et al., 2022).

In this study, we used human models to avoid the caveats of interspecies limitations which are especially marked in the fields of ZIKV infection, GBM biology and myeloid identity states (Gorman et al., 2018; Oh et al., 2014; Sankowski et al., 2019). For example, infection of mouse central nervous system cells typically requires a genetic background of *Ifn1b* loss-of-function, and/or mouse adapted virus strains (Gorman et al., 2018; Lazear et al., 2016). While we cannot exclude some degree of *in vitro* artefact, there is ample clinical evidence for high infectability of HDB, captured faithfully in our primary slice culture experiments. It is therefore reasonable to assume that culture conditions do not materially impair tissue permissivity to ZIKV in GBM. In fact, over time in slice culture we would expect SOX2+ glioma cells to outgrow the differentiated myeloid fraction, and perhaps become more susceptible to Zika, yet within the timeframe of our experiments (up to 7 days) we did not observe such a tendency. Instead we found that SOX2+ progenitors in GBM were refractory to ZIKV infection relative to HDB SOX2+ progenitors, and that this property derived from non-contact mediated effects of myeloid cells.

### A common myeloid pathway regulates ZIKV infection of human developing brain and malignant neural progenitors

Our findings indicate the myeloid cell secretome regulates resilience against ZIKV infection of developing and malignant human neural progenitors. While such progenitors share a common transcriptional identity (Bulstrode et al., 2017; Ligon et al., 2007; Suvà et al., 2014), we observed that HDB tissue was markedly more permissive to ZIKV infection compared to GBM. While GBM virus RNA levels in this study were similar to those previously reported (~1000-1500 viral genome copies per ng RNA) (Zhu et al., 2020), direct comparison to HDB tissue in this study reveals the dramatic relative extent of refractory ZIKV infection in GBM. Interrogating tropism at the single cell level indicated only mildly enhanced infection of proneural (neural progenitor cell-like and oligodendrocyte precursor cell-like) glioma cell subtypes compared to astrocyte-like and mesenchymal-like glioma cells (Neftel et al., 2019). Across the ZIKV-mCh+ GBM single cells analysed, the presence of SOX2 transcripts was associated with higher ZIKV read counts, in keeping with an established role for SOX2 in driving ZIKV target cell identity (Zhu et al., 2020). Conversely, we found a strong correlation of HDB and GBM sample myeloid cell signatures with ZIKV resistance. Whereas the few myeloid cells present in HDB can themselves support ZIKV infection (Retallack et al., 2016; Wang et al., 2018; Xu et al., 2020), we found that GBM myeloid cells do not allow the virus to reproduce, as previously reported for oncolytic herpes simplex virus (Delwar et al., 2018). We speculate that this difference could reflect either functional immaturity of myeloid cells in the developing brain, or cell origin, since bone marrow derived macrophages rather than brain microglia can represent the majority myeloid population in GBM (Buonfiglioli and Hambardzumyan, 2021). Importantly, our study highlights the contribution of the myeloid secretome in regulation of GBM resistance to ZIKV. We observed that GBM myeloid cells secreted a variety of both pro- and anti-inflammatory cytokines, which may reflect a diversity of activation states at baseline. However, single cell analysis demonstrated that ZIKV-mCh+-selected myeloid cells converged on primarily pro-inflammatory anti-viral transcriptomic signatures (Sankowski et al., 2019).

### Myeloid secretion of IFNβ renders GBM refractory to ZIKV

In keeping with prior studies addressing stem cell antiviral responses (Burke et al., 1978; Wu et al., 2018, 2019), we found that GBM and HDB neural progenitors exhibited limited capacity to produce interferons themselves in response to exposure to viral mimetic poly(I:C). Treatment with exogenous recombinant IFNβ (or myeloid conditioned medium) conferred ZIKV resistance as well as a signature of downstream JAK-STAT signaling in GBM lines. The JAK1/2 inhibitor ruxolitinib (Quintás-Cardama et al., 2010) almost completely restored ZIKV infection in GBM lines despite treatment with myeloid cell conditioned media (11b+CM) or IFNβ, whereas in HDB lines, ruxolitinib-induced rescue was incomplete, suggesting that other cytokines present could also be involved. Therapeutic targeting of the myeloid cell secretome could present clinically treatment possibilities in conjunction with ZIKV (Pyonteck et al., 2019). For example, our study suggests enhanced killing of GBM progenitors by ZIKV when given with inhibitors of Type 1 interferon (e.g., IFNAR1 blocking antibody anifrolumab (Peng et al., 2015), TYK2 inhibitor deucravacitinib (Papp et al., 2018) and/or inhibitors of JAK-STAT signaling such as ruxolitinib (Quintás-Cardama et al., 2010), which could be delivered with virus locally to the tumour resection cavity to minimise systemic side effects.

### HDB neural progenitors can be protected against ZIKV by the myeloid secretome or treatment with IFNβ

Our results agree with prior findings that myeloid cells in HDB are sparsely distributed and morphologically immature in first trimester HDB, coinciding with the greatest vulnerability to Zika Congenital Syndrome (Andjelkovic et al., 1998). We speculate that the paucity and/or functional immaturity of myeloid cells at early stages of human brain development could underlie devastating congenital neuropathology due to ZIKV in particular and TORCH infections more widely, but further research is needed (Megli and Coyne, 2021). Our findings indicate that developing and malignant neural progenitors depend on myeloid cell-secreted type 1 interferons for effective ZIKV resistance. RNA-Seq data revealed no differential expression of neural ‘stemness’ markers after myeloid cell-CM treatment in either HDB or GBM lines, indicating that myeloid cells can induce a potent anti-viral response in these populations without changing their identity or progeny outputs. Indeed, Type 1 interferon is administered clinically for treatment of viral hepatitis, myeloproliferative disorders and multiple sclerosis among others, and available data in women exposed during pregnancy suggests no increased incidence of adverse fetal outcomes (Hellwig et al., 2020; Zhang et al., 2021)

## Star Methods

### Resource Availability

#### Lead contact

Further information and requests for resources and reagents should be directed to and will be fulfilled by the lead contact, David Rowitch (dhr25@medschl.cam.ac.uk).

#### Materials availability

This study did not generate new unique reagents.

## Experimental Model And Subject Details

### Primary Human Tissue (Table S2 and S3)

Patient GBM tissue was obtained with informed consent under UK Health Research Authority permissions REC 18/WM/0094 (Principal Investigator Harry Bulstrode), or REC 18/EE/0172 (Principal Investigator Richard Mair) and processed immediately following resection. Patient demographics and routine histopathological data are provided in Table S2. The acquisition of HDB tissue was undertaken by Xiaoling He under provisions of UK Health Research Authority REC 96/085 (Principal Investigator Roger Barker). Gestational age data is provided in Table S3, but no information on sex was available at these early developmental timepoints. Both patient GBM tissue and HDB tissue were processed under the provisions of REC 18/WM/0094 and in accordance with UK Human Tissue Authority regulations, and informed consent was obtained in all cases. GBM and HDB samples were either processed for dissociated cell culture and/or slice culture. In addition, small pieces of the sample were immediately fixed in 4% paraformaldehyde (PFA) overnight at 4 °C for later IF as T0 tissue, or snap frozen on OCT at -80 °C for nuc-RNA seq.

### Primary GBM and HDB slice culture

Diced tissue pieces 2-3 mm square were mounted in 4% low melting point agarose in DMEM. 350 μm slices were cut using a Leica VT1200 Vibratome then transferred onto 0.4 μm organotypic inserts (Millipore/Sigma-Aldrich, PICMORG50) in a 6 well plate containing 1ml per well of Slice culture maintenance media - Neurobasal (Life Technologies, 21103049) supplemented with 10mM HEPES, 1x B-27 supplement, 1x Penicillin-Streptomycin, and Nystatin (6 U/mL, Sigma-Aldrich, N1638). The plate was then incubated for 3-5 days at 37 °C with regular media changes before ZIKV infection.

### Primary Dissociated Adherent Cell Culture

Tissue was diced in HBSS (Thermo Fisher Scientific, 14170112), spun for 5 min at 300*g*, and resuspended in activated Papain (Lome, LS003126) in Hibernate A media (Thermo Fisher Scientific, A1370501). After 20 min (for HDB) or 45 min (for GBM) incubation at 37 ºC, the tissue was spun down, and resuspended in isolation media (Hibernate A supplemented with FBS, 1x B-27, Insulin (Sigma-Aldrich, I9278, 4g/ml) and Sodium pyruvate (220g/ml)). The digested tissue was triturated using a glass pipette filtered over a 50 μm cell strainer into a 10% Percoll gradient (Sigma-Aldrich, GE17-5445-01) then spun for 20 min at 800 rcf (soft). The pellet was resuspended in 1ml red cell lysis buffer for 90 seconds. The reaction was stopped with DMEM/F-12 and spun for 5 min at 300g. Cells were either plated in flasks in NSC media or used for magnetic-activated cell sorting. Cells were either replated to 12 or 24 well plates for Zika infection up to 7 days post isolation, or frozen in NSC media + 10% DMSO 1-3 days after isolation.

NSC media was used for all dissociated cell culture, primary and GBM lines: DMEM/F12 (Gibco, 11320-033), supplemented with 15mM HEPES (Stock 1M, Sigma-Aldrich, H0887), D-Glucose (7.25ml of 100 g/L, Sigma, G8644), 1x MEM-NEAA (LifeTech/Gibco, 11140-035), 1x Pen-Strep (LifeTech/Gibco, 15140-122), BSA (800 μL of 7.5% LifeTech/Gibco, 15260-037), bMercETOH (100 μM, LifeTech/Gibco, 31350-010 (20ml), B27 (0.5x, LifeTech/Gibco, 17504-044), N2 (0.5x, LifeTech/Gibco, 17502-048), EGF (10ng/ml, Peprotech, 315-09-500), bFGF (10ng/ml, Peprotech, 100-18B-500), Laminin, (1μg/ml, Cultrex, 3446-005-01). Glass plates were pre-coated with poly-L-ornithine (PLO) solution in PBS (10-15 μg/mL) at room temperature for 2 h. PLO solution was removed, plates washed twice with PBS, followed by a third wash with DMEM/F-12. Laminin was diluted in NSC media (20 μg/mL), added to PLO-coated glass plates or to plastic flasks/plates without PLO coating, and incubated at room temperature (15 – 25 °C) for at least 2 hours, then removed and fresh media added.

### Cell Lines (Table S4)

GBM and HDB cell lines were generated and supplied from the Glioma Cellular Genetics Resource (gcgr.org.uk) with ethical approval from the NHS Health Research Authority (East of Scotland Research Ethics Service, REC reference 15/ES/0094). DMG cell lines were kindly provided by Angel Carcaboso and Chris Jones as indicated (Table S4). Cell lines were cultured in NSC media on laminin coated culture-ware at 37C. Medium was changed every 3 days and cells were passaged using StemPro Accutase Cell Dissociation Reagent (Life Tech, A1110501). Cells were tested for mycoplasma regularly. No additional authentication was conducted on receipt of cell lines from the sources detailed.

## Method Details

### Magnetic-activated cell sorting (MACS isolation)

To isolate myeloid cells from the primary bulk GBM single cell suspension, MACS was used following the manufacturer’s instructions. A concentrated cell suspension was incubated with anti-CD11b conjugated magnetic beads (1:50, Miltenyi 130-049-601) for 15 min at 4 °C. Unbound beads were removed by dilution and spun down, then the cells resuspended in 1ml of Hibernate-A and passed through an MS column (Miltenyi, 130-042-201) on a magnetic stand. The flow-through was collected as the GBM11b-fraction. The column-bound GBM11b+ population were subsequently eluted into a separate tube using Hibernate-A.

### ZIKV preparation

ZIKV PE243 stocks were generated by transfecting PE243 RNA (supplied by A. Kohl, Centre for Virus Research, University of Glasgow, Lindomar J. Pena and Rafael Oliveira de Freitas França, Fiocruz Recife, Pernambuco, Brazil) into Vero 2-2 cells using Lipofectamine 2000 (Life Technologies). Zika PE243 virus was amplified inoculating 100 μL of the rescued virus in 80-90% confluent flasks of Vero 2-2 cells. After 2-3 days, infected cell supernatants were pooled, centrifugated 10 min at 1,300 × *g*, 4°C and filtered through a 0.22μm membrane. The pooled supernatant was supplemented with 10% glycerol then aliquoted in cryovials and stored at -80°C. ZIKV-mCh production and titration was performed as previously described (Fajardo et al., 2020), based on a plasmid very generously shared by Andres Merits (Mutso et al., 2017), modified to incorporate T7 in place of SP6 promoter, also as previously described (Fajardo et al., 2020).

### ZIKV titration

Plaque forming assay was used to calculate viral titers (plaque forming units (PFU)/mL). Vero 2-2 cells were seeded in 6 well plates at density of 1.5 × 10^5^ cells/well and incubated at 5% CO2, 37 °C for 48 hours before infection. Serial dilutions of the viral stocks were made and then added to the Vero cells for 1 hour. Cells were covered with 2 mL Carboxy Methyl Cellulose overlay solution (CMC/DMEM) and further incubated for 5-7 days. CMC/DMEM overlay was washed with PBS and infected cells were fixed and stained for 30 min using a solution of crystal violet containing paraformaldehyde for plaque visualisation.

### ZIKV infection

glioma cells and primary glioblastoma cells were seeded in 12 or 24 well plates at densities of 3x10^5^ or 1.5x10^5^ cells/well respectively and allowed to attach overnight. For later immunofluorescence, cells were plated in 8 well chamber slides, at a density of 5x10^4^ cells/well, or μ-clear imaging plates. For RNAscope, cells were plated into multiwell chamber slides with a removable chamber (IBIDI or Thermofisher Scientific, 177445PK). Cells were exposed to ZIKV (MOI:1 for all GBM or HDB cells or MOI:3 for primary GBM cells; MOI 10 for ZIKV-mCh, all cell types) for 2 h at 37 ºC, washed with PBS and maintained for 48 or 72 h. Cells for MOCK infection were seeded in separate plates and processed in parallel.

Brain tumour tissue slices were maintained in PTFE inserts in 6 well plates. Virus addition was performed by dispensing 200 μL inoculum onto the air-facing surface of the slice, with the remaining 800 μL inoculum into the well under the insert. Total inoculation was 1×10^7^ PFU of ZIKV, with adsorption period of 4 hours, then inserts were washed with PBS and transferred to a maintenance plate. Media was changed every 48h by transferring PTFE inserts to new equilibrated maintenance plates. After 3 or 7 days, slices were either transferred into RNAlater RNA stabilization Reagent (ThermoFisher, AM7020) for ZIKV RNA quantification by RT-qPCR, or fixed in 4% formaldehyde for 4 hours at RT or overnight at 4 °C for IF or smFISH.

### Conditioned Media collection and experiments

Conditioned media was harvested from GBM cell lines, GBM11b- and GBM11b+ cells cultured in 200,000 cells per ml of media, 48 hours post plating, to generate LineCM, 11b-CM and 11b+CM conditioned media stocks respectively. Where indicated, cells/media were also supplemented with 10 μg/ml poly(I:C) (InvivoGen, tlrl-pic) during conditioning, also for 48 hours total, to generate Pi:c-LineCM, Pi:c-11b-CM and Pi:c-11b+CM conditioned media stocks respectively. 0.22 μm syringe filters were used to remove cell and debris contamination at the time of harvesting. Media was frozen in aliquots at -80 °C if not used immediately.

For RT-qPCR analysis of ZIKV infection following CM treatment (Figure 6B), cells were incubated in CM without poly(I:C) diluted 50:50 with NSC media for 12h before infection, and for 48h after infection.

For conditioned media RNA-Seq experiments (Figure 6C; Figure S5E and F), GBM E22 or GBM E34 cells were plated in a 24 well plate and incubated in 150 μl CM + 250 μl NSC media for 48 h. Individual RNA-Seq libraries were prepared using 11b+CM from 3 separate patient GBM11b+ fractions and 3 LineCM controls.

### IFNβ and ruxolitinib Experiments

For the IFNβ dose response on ZIKV infection (RT-qPCR analysis), GBM lines were incubated in NSC media supplemented with appropriate doses of IFNβ (R&D Systems, 8499-IF-010) 24 h before ZIKV infection and after viral adsorption until RNA harvesting 48 h after infection.

For the assessment of JAK/STAT signalling by Western, GBM lines in 6 or 12 well plates were incubated in NSC media or CM media supplemented with appropriate doses of IFNβ or ruxolitinib (Selleck Chemicals, S1378) for 3 h. Then the cells were washed 1x in cold DPBS and harvested in 1x sample buffer with protease and phosphatase inhibitors (Fisher Scientific, 78441).

For ruxolitinib and IFNβ rescue experiments in GBM E22 cells, base media or CM were supplemented with ruxolitinib at the indicated concentrations. Cells were pre-treated with CM or base media +/-recombinant IFNβ, +/-ruxolitinib, for 24 h prior to ZIKV infection, then grown in unsupplemented base media for 48 h prior to fixation/lysis for assay of productive infection.

For IFNβ treatment combined with ZKV infection in HDB slice culture, 3 slices were left uninfected (MOCK), 3 were infected with ZIKV as detailed above, and 3 were pretreated with 100 pg/mL IFNβ for 24 h before ZIKV infection. Slices were fixed at 72 h.p.i. and processed for IF.

### Western blotting

0.3-2 million cells were pelleted, washed once in cold PBS, spun down, the cell pellet aspirated dry and frozen on dry ice. Proteins were extracted in RIPA buffer and protein concentration quantified using a BCA assay or a Direct Detect Spectrometer. For the STAT-1 Westerns, cells growing in 12 or 6 well plates were washed in cold PBS, then sample buffer added directly to the cells at a ratio of 5 × 10^6^ cells/ml sample buffer. The cells were then scraped off the plate, transferred straight to tubes on ice, and frozen at -80 °C until use. The lysate was thawed on ice, heated for 5 min at 95 °C and, vortexed briefly, spun at 14000rpm for 8 min at 4 °C and the supernatant collected. For western blots, extracts were separated by SDS-PAGE on 4-12% Bis-Tris NuPage gels (Invitrogen) and blotted onto nitrocellulose membrane before blocking for 1 h with Intercept (TBS) blocking buffer (LI-COR). All antibodies (Table S5) were diluted in 50:50 Intercept blocking buffer: TBS-tween (TBST 0.1%). Primary antibodies were incubated on the blots overnight at 4 °C. Blots were washed in TBST, then fluorescent conjugated antibodies (LI-COR, 1:5000) added at room temperature for 2 h. Finally, blots were washed in TBST and imaged using the LI-COR Odyssey system.

### Immunofluorescence (IF) for dissociated adherent cells

Cells were fixed using 4% paraformaldehyde (PFA) for 10 min, then washed thoroughly with PBS. Cells were permeabilized with PBS containing 0.5% Triton-X-100 for 5 min, then blocking solution added (PBS with 0.5% bovine serum albumin (BSA), 0.1% Triton X-100) for at least 1 hour but up to 2 days. All antibodies are listed in Table S5. Primary antibodies were added in blocking solution for 2 h at 37 °C, or overnight at 4 °C. Cells were then washed three times for 10min with PBST (PBS with 0.1% Triton X-100). Cells were stained with an appropriate secondary antibody (Alexa Fluor 488, 594 or 647; 1:500, Invitrogen) for 1 h at room temperature. Cells were washed twice for 5 min with PBST, once with PBS, incubated in DAPI for 10 min (1:5,000 in PBS), then washed with PBS and imaged either in the plate on a Leica SP5 confocal at 20x or 40x or Operetta CLS at 20x.

### Immunofluorescence for GBM and HDB slices

After fixation slices or T0 tissue were washed 3 times >10 min in PBS. Samples were placed in 20% sucrose for cryoprotection for 24–48 h at 4 °C, then embedded in optimal cutting temperature (OCT) compound, frozen in an ethanol/dry ice bath and stored at –80 °C. 14-16 μm cryosections were cut onto superfrost slides using a CM3050S cryostat (Leica Microsystems) and stored at –80 °C until IF staining or smFISH. For IF, slides were allowed to equilibrate at RT in PBS for 15 min, then permeabilised with PBS containing 1% Triton-X-100 for 15 min. Blocking solution was then added for 1 h, and the slides incubated in primary antibodies in blocking solution overnight at 4 °C. Sections were then washed four times for 15 min with PBST, and appropriate secondary antibodies added (Alexa Fluor 488, 594 or 647; 1:500, Invitrogen) in blocking solution for 1.5-2 h at room temperature. Cells were washed twice for 15 min with PBST, once with PBS, incubated in DAPI for 10 min, then washed with PBS and coverslips applied using Prolong Gold or Diamond.

### smFISH (RNAscope) sample preparation

HDB or GBM tissue sections were stored at -80 °C and transferred directly to ice-cold 4% paraformaldehyde (PFA) solution for 45 min. Slides then underwent citrate buffer (1x, pH 6.0, Sigma-Aldrich, C9999) heat-induced antigen retrieval at 95 °C for 15 min. Slides were then washed and dehydrated in PBS (1X) and ethanol gradients from 50% to 100% for a total of 30 min. Slides were air-dried before automated single molecule fluorescent in situ hybridization (smFISH) protocol. Cell cultures adherent to plastic or positively charged glass slides were fixed in 4% PFA for 45 min, dehydrated in ethanol gradients and stored at -20 °C in 100% ethanol for ~ 2 weeks. Slides were rehydrated in ethanol gradients from 100% to 50% directly before the automated smFISH protocol.

Multiplex smFISH was performed on a Leica BondRX fully automated stainer, using RNAScope© Multiplex Fluorescent V2 technology (Advanced Cell Diagnostics, 322000). GBM tissue section slides underwent heat-induced epitope retrieval (HIER) with Epitope Retrieval Solution 2 (pH 9.0, Leica AR9640) at 95 °C for 10 min. HDB tissue sections underwent the above HIER for 5 min, and adherent cells did not undergo heat treatment. GBM, HDB tissue and cells were then incubated in RNAScope© Protease III reagent (ACD 322340) at 42 °C for 15, 10 and 5 min, respectively. All slides were then treated with RNAScope© Hydrogen Peroxide (ACD 322330) for 10 min at RT to inactivate endogenous peroxidases. All double-Z mRNA probes were designed against human genes by ACD for RNAScope© on Leica Automated Systems and are listed in table S6. Further information is readily available from the manufacturer (https://acdbio.com/catalog-probes).

Slides were incubated in RNAScope 2.5 LS probes for 2 h at RT. DNA amplification trees were built through consecutive incubations in AMP1 (preamplifier, ACD 323101), AMP2 (background reduction, ACD 323102) and AMP3 (amplifier, ACD 3231003) reagents for 15 to 30 min each at 42 °C. Slides were washed in LS Rinse buffer (ACD 320058) between incubations.

After amplification, probe channels were detected sequentially via HRP–TSA labelling. To develop the C1–C3 probe signals, samples were incubated in channel-specific horseradish peroxidase (HRP) reagents for 30 min, TSA fluorophores for 30 min and HRP-blocking reagent for 15 min at 42 °C. The probes in C1, C2 and C3 channels were labelled using Opal520 (Akoya FP1487001KT), Opal570 (Akoya FP1488001KT), and Opal 650 (Ak oya FP1496001KT) fluorophores (diluted 1:500). The C4 probe complexes were first incubated with TSA–Biotin (Akoya NEL700A001KT, 1:250) for 30 min at RT, followed by streptavidin-conjugated Atto425 (Sigma 56759, 1:400) for 30 min at RT. Samples were then incubated in DAPI (Sigma, 0.25μg /ml) for 20 min at RT, to mark cell nuclei. Slides were briefly air-dried and manually mounted using ~90 μl of Prolong Diamond Antifade (Fisher Scientific) and standard coverslips (24 × 50 mm^2^; Fisher Scientific). Slides were dried at RT for 24 h before storage at 4 °C for >24 h before imaging.

### smFISH and IF automated imaging and analysis

#### Slice culture imaging and analysis

Slides and plates were imaged on either a Leica SP5 confocal or an Operetta high-content imaging system (Perkin-Elmer) with Harmony software, settings as detailed in Table S7. Image analysis was carried out using Fiji, Python, or Harmony 4.9 software (Perkin Elmer). Data and statistical analyses were carried out in Excel or GraphPad Prism. Brightness and contrast were adjusted for display, always to the same levels when comparing across tissues.

To locate whole-tissue sections or regions of interest for high-resolution imaging, entire slides were initially scanned under low magnification using a ×5 numerical aperture (NA) 0.16 objective (pixel size: 7.2 μm). Regions of interest for ×40 scans were manually selected on low-magnification previews. Selected ×40 fields were imaged with an 8% overlap. The high-resolution smFISH images of cells and tissue sections were acquired on the spinning-disk confocal mode using a sCMOS camera and a ×40 NA 1.1 automated water-dispensing objective. Each field was imaged as a *z*-stack consisting of 20 planes, with a 1 μm incremental step size.

To segment single nuclei, myeloid cells, progenitors and *ZIKV*+ cells, analysis scripts were created in Harmony 4.9 using customisable building block functionality. To identify single nuclei, Gaussian blurred DAPI images were segmented using intensity, size, and contrast thresholds. To identify *RUNX1*+/*CD68+* myeloid cells and *SOX2*+ progenitor cells, a peri-nuclear region was created by increasing the area of each nucleus by 3 μm^2^. *RUNX1, CD74* and *SOX2* mRNA puncta were then quantified within the nucleus (+3 μm^2^) and cell populations were selected based on spot number, intensity, size, and contrast thresholds. To analyse *ZIKV* in HDB tissue sections, *ZIKV*+ cells were identified by segmenting the cytoplasm around the nuclei based on *ZIKV* signal intensity thresholding. To analyse *ZIKV* in GBM tissue and cell cultures, *ZIKV*+ regions were segmented based on *ZIKV* signal intensity thresholding and morphology characteristics. *ZIKV*+ nuclei were then segmented within these regions based on Gaussian smoothed DAPI intensity characteristics. Cell populations were quantified as a ratio of total nuclei analysed.

To identify IBA1 and SOX2+ cells in TO sections, the background and autofluorescence of the tissue was corrected by subtracting the image of an empty channel. SOX2+ cells were then selected based on SOX2 signal intensity thresholding. IBA1 + image regions were identified in the tissue, and IBA1+ cells were identified based on both nuclear overlap of at least 30% with these regions and IBA1 nuclear signal intensity thresholding.

To identify SOX2 and ZIKV positive cells after IF staining in HDB tissue, nuclei and cell cytoplasm were segmented in Harmony. SOX2+ cells were selected based on nuclear SOX2 signal intensity thresholding and ZIKV+ cells selected based on ZIKV intensity thresholding in the whole cell. Double SOX2+ and ZIKV+ cells were counted and expressed as a percentage of the SOX2+ cells. 4-30 thousand SOX2+ cells were analysed per condition, across 3 slices per condition, 1-3 replicate cryosections per slice, in 1 HDB sample.

#### Dissociated adherent cell imaging and analysis

For counting SOX2 or IBA1 positive cells representative fields of mock infected cultures were imaged at 20 or 40x on the confocal, then image processing and nuclei counting was implemented with a python script using the scikit-image library. First, background and flatfield correction was applied to the input image (I) based on Gaussian (sigma=5) blurred input image (GI). The corrected image was then calculated using the equation: I/(GI/max(GI)) - 0.3*GI. Afterwards, multiotsu thresholding and watershed algorithm was employed to segment the nuclei on different channels (both DAPI and SOX2). Among the segmented regions, SOX2 positive nuclei were then picked out if the overlapping area of the regions from two channels was greater than 80% of that in the DAPI channel.

IBA1 positive cells were either counted manually or by nuclear signal intensity thresholding.

For counting SOX2 and IBA1 positive cells imaged on the Operetta CLS, Harmony 4.9 software was used. To identify single nuclei, Gaussian blurred DAPI images were segmented using intensity, size, and contrast thresholds. SOX2 or IBA1 cell populations were selected based on nuclear intensity thresholds.

To calculate the average proportion of SOX2 and IBA1 positive cells in the GBM, GBM11b- and GBM11b+ fractions, cell counts were performed from at least 3 separate GBM samples for each fraction.

### Zika viral RNA quantification

RNA was extracted from cells using the RNeasy® Mini Kit (Qiagen) with DNAse treatment. Brain slices stored in RNAlater were disrupted and homogenised using the TissueRuptor® II (Qiagen) and RNA extracted with the RNeasy® Lipid Tissue mini Kit (Qiagen) following the manufacture’s protocol. Total RNA was then quantified by NanoDrop 8000 quantification (Thermo Fisher Scientific). RT-qPCR was carried out using TaqMan® chemistry, with a commercial quantification kit (Genesig Standard Zika Virus Quantification Kit, PrimerDesign), using One-Step 2X RT-qPCR Reagent (PrimerDesign).

### RNA extraction, library preparation and sequencing

For all samples, RNA was extracted using Qiagen RNeasy Micro Plus kit and analysed on Bioanalyser to confirm RIN >8. Library preparation was undertaken using NEB Ultra II Directional Kit (Poly A enrichment). For primary cell ZIKV-mCh sorted fractions with smaller cell numbers and lower input RNA concentrations, Takara Smartseq pico V2 library preparation (Ribosomal depletion) was used. In each case library preparation was performed according to manufacturers’ instructions. Paired end sequencing was performed using Illumina Novaseq PE50.

### Bulk RNA-Seq Analysis

Quality checks on the raw fastq files were performed using FastQC (v0.11.8) (Andrews, 2010), summarised with MultiQC (v1.9) (Ewels et al., 2016). The fastq files were subsampled (to 50M reads and 25M reads for the mCherry and myeloid cell experiment respectively) to achieve a uniform sequencing depth (Mohorianu et al., 2017). For the mCherry experiment, the first 3 nucleotides for both forward and reverse reads were trimmed using TrimGalore (v0.6.4_dev, based on cutadapt v3.4) (Kruger, 2012) to account for low sequence quality. The resulting fastq files were aligned to the *H. sapiens* reference genome (build GRCh38.p13) using STAR (v2.7.0a) with default parameters (Dobin et al., 2013) The count matrices were generated using featureCounts (subread package, v2.0.0) (Liao et al., 2014).

Further quality checks on the count matrices included density and violin plots, illustrating the abundance distributions across samples, MA plots for pair-wise comparisons of replicates focusing on the per-gene fold-differences across abundances. In addition, Jaccard Similarity Index (JSI) heatmaps show the robustness of the most highly expressed genes across samples, dendrograms exemplify the hierarchical similarity in transcriptomic signatures and Principal Component Analysis (PCA) plots present the inter-sample relationships under a dimensionality reduction, to assess similarity.

A noise analysis was performed using noisyR (v1.0.0) (Moutsopoulos et al., 2021) to correct for artificially high fold-changes exhibited by low expression genes. The noise threshold, added to each expression matrix, was calculated to be 300 for the mCherry experiment and 15 for the myeloid cell experiment, with ~19k and ~23k genes kept for further analysis respectively. A quantile normalisation (Bolstad et al., 2003) was then applied on the denoised matrices.

Differential expression (DE) analysis was performed using both edgeR (v3.34.0) (McCarthy et al., 2012) and DESeq2 (v1.32.0) (Love et al., 2014). The comparisons focused on GBM_HR vs GBM_MR and Zika positive vs negative for the mCherry experiment, and control vs myeloid cell media type for the myeloid cell experiment. Due to the similarity of the two conditions for individual cell types in the myeloid cell experiment, DE was performed both on each cell type individually (GBM E22, GBM E34, HDB) and on the whole dataset.

An enrichment analysis was performed on all DE genes in each comparison (for robustness the intersection between edgeR and DESeq2 was considered; the two packages largely agreed after the noise correction (Moutsopoulos et al., 2021), with all genes expressed in each dataset (with maximum expression higher than the noise threshold) as background using the g:profiler (https://biit.cs.ut.ee/gprofiler/gost) R package (v0.2.0) (Raudvere et al., 2019) and Reactome datasets (Viteri et al., 2019).

For the ZIKV-mCh experiment, the genes potentially regulated by ZIKV were identified by splitting the viral genome into 30 nucleotide windows (with 20nt overlap) and aligning them to the *H. sapiens* reference genome (build GRCh38.p13) using STAR (v2.7.0a) with default parameters (Dobin et al., 2013) The genes proximal to the aligned coordinates were then identified.

### CIBERSORT Analysis

The bulk RNA-Seq libraries were submitted to the CIBERSORT analysis pipeline (Newman et al., 2015) in absolute mode using the LM22 gene signature file, permutations = 500, quantile normalisation disabled. The 22 gene types incorporated in this scheme were collapsed for display purposes to the 9 gene types displayed by summation of the absolute scores for relevant subtypes. For example, plotted ‘B-cells’ score represents the sum of absolute scores for B-cell naïve, B-cell memory and Plasma cell LM22 categories. Likewise ‘T-cells’, ‘NK cells’, ‘myeloid cell’, ‘Dendritic cells’, ‘Mast cells’ are each composite terms representing the sum of the relevant subtype scores.

### Single Cell RNA-Seq Analysis

#### Plate-based scRNA-seq (ZIKV mCh+ libraries)

Plate based scRNA-seq was performed with the NEBNext Single Cell/Low Input RNA Library Prep Kit for Illumina (New England Biolabs Inc, E6420L). Briefly, single cells were sorted into a pre-prepared 384-well plate (Eppendorf, Cat. No. 0030128508) or 96-well plate (Eppendorf, Cat. No. 0030128648) containing 2 μl of 1X NEBNext Cell Lysis Buffer. Sorted single cells were sealed and spun at 100 x g for 1 minute then immediately frozen on dry ice and stored at -80 °C.

cDNA generation was then performed in an automated manner on the Agilent Bravo NGS workstation (Agilent Technologies). Briefly, 1.6 μl of Single Cell RT Primer Mix was added to each well and annealed on a PCR machine (MJ Research Peltier Ther-mal Cycler) at 70°C for 5 minutes. 4.4 μl of Reverse Transcription (RT) mix was added to the mixture and further incubated at 42°C for 90 minutes followed by 70°C for 10 minutes to generate cDNA. 22 μl of cDNA amplification mix containing NEBNext Single Cell cDNA PCR MasterMix and PCR primer was mixed with the cDNA, sealed and spun at 100 x g for 1 minute. cDNA amplification was then performed on a PCR machine (MJ Research Peltier Thermal Cycler) with 98°C 45 s, 20-25 cycles of [98°C 10 s, 62°C 15 s, 72°C 3 mins], 72°C 5 mins.

The plate containing the amplified cDNA was purified with an AMPure XP workflow (Beckman Coulter, Cat No. A63880) and quantified with the Accuclear Ultra High Sensitivity dsDNA kit (Biotium, Cat. No. 31028). ~10 ng of cDNA was stamped into a fresh plate for sequencing library preparation.

Sequencing libraries were then generated on the Agilent Bravo NGS workstation (Agilent Technologies). Purified cDNA was fragmented by the addition of 0.8 μl of NEB-Next Ultra II FS Enzyme Mix and 2.8 μl of NEBNext Ultra II FS Reaction buffer to each well and incubated on a PCR machine (MJ Research Peltier Thermal Cycler) 72°C 15 mins, 65°C 30 mins. A ligation mixture was then prepared containing NEBNext Ultra II Ligation Master Mix, NEBNext Ligation Enhancer and 100 μM Illumina compatible adapters (Integrated DNA Technologies) and 13.4 μl added to each well of the plate. The ligation reaction was incubated on the Agilent workstation at 20°C for 15 minutes and then purified and size selected with an AMPure XP workflow (Beckman Coulter, Cat No. A63880).

20 μl of KAPA HiFi HS Ready Mix (Kapa Biosystems, Cat. No. 07958927001) was then added to a pre-prepared plate (Eppendorf, Cat. No. 0030128508) containing 100 μM i5 and i7 indexing primer mix (50 μM each) (Integrated DNA Technologies). The indexing primers pairs were unique to allow multiplexing of up to 384 single cells in one sequencing pool. The plate containing the PCR Master Mix and indexing primers was stamped onto the adapter ligated purified cDNA, sealed and spun at 100 x g for 1 minute. Amplification was performed on a PCR machine (MJ Research Peltier Thermal Cycler) with 95°C 5 min, 8 cycles of [98°C 30 s, 65°C 30 s, 72°C 1 min], 72°C 5 mins. The PCR products were pooled in equal volume on the Microlab STAR automated liquid handler (Hamilton Robotics) and the pool purified and size selected with an AMPure XP workflow (Beckman Coulter, Cat No. A63880). The purified pool was quantified on an Agilent Bioanalyser (Agilent Technologies) and sequenced on one lane of an Illumina HiSeq 4000 instrument (75bp paired end, HiSeq 4000 150 cycle kit).

#### Preprocessing

10X v2 single-nucleus RNAseq data was aligned to human genome version GRCh38 using the cellranger software and processed with the CellBender (Fleming et al., 2019) algorithm before any further downstream analysis to remove ambient RNA. SmartSeq data was aligned to human genome version GRCh38 augmented for the ZIKV genome with the STAR algorithm (Dobin et al., 2013). We further used the scrublet algorithm (Wolock et al., 2019) to remove doublets, manually choosing doublet score thresholds for each sample, based on the bimodal histograms of doublet scores.

#### Quality Control

We plotted QC plots using number of detected genes, number of counts and percent of counts coming from mitochondrial genes and removed outlier cells. For the SmartSeq data removed cells had less than 2500 detected genes, more than 12000 detected genes, less than two million reads or more than 10% of reads coming from mitochondrial genes. For the 10X data removed cells had less than 500 detected genes, more than 5000 detected genes, less than 400000 counts or more than 10% of counts for mitochondrial genes.

#### Calculation of glioma cells subtype scores

We then calculated glioma cell subtype score according to methods previously outlined (Neftel et al., 2019) Briefly, for genes i and sample j, this meant considering only genes with E_a_ > 4, where E_a_ is given by E_a_(i) = log_2_(average(TPM_i,1…n_)+1). Furthermore, expression levels were defined as E_i,j_ = log_2_(TPM_i,j_/10+1), based on which relative expression levels were defined as Er_i,j_ = E_i,j_-average[E_i,1…n_]. These relative expression levels, were than used to calculate gene signature scores, as SC_j_(i) = average[Er(G_j_,i)] – average[Er(G_j_^cont^,i), where Er(G_j_,i) is the average relative expression of genes included in the expression signature of interest (mesenchymal cell-like, oligodendrocyte precursor cell-like, astrocyte-like, neural progenitor cell-like), obtained from Extended Data Table 2 in the original publication (Neftel et al., 2019) and Er(G_j_^cont^,i) is a control gene set. This control gene set was defined as in the original publication, by binning all genes into 30 groups based on their expression and then selecting for each gene in the gene signature score 100 control genes of similar aggregate expression.

#### Normalisation and clustering

We used the scanpy python package (Wolf et al., 2018) to normalise and log-transform the data, as well as regress out the effects of total counts and mitochondrial counts. We then used the BBKNN algorithm (Polański et al., 2019) for batch alignment followed by Louvain clustering.

#### Classification of primary cell types

We visualised the expression of brain cell type markers, as well as glioma cell subtype scores for each cluster in a heatmap. Based on this we classified a cell as non-malignant if it displayed distinct expressed of typical markers of a brain cell type (e.g., myeloid cell, astrocytes, excitatory neuron) and had low glioma cell subtype scores. Similarly, we classified a cell as glioma cell if it had a high expression of glioma cell subtype scores and expressed indistinct expression of multiple cell type markers.

#### Classification of glioma cell subtypes

We recalculated the glioma cell subtype scores only on the subset of glioma cells, so we excluded non-malignant cells, such as myeloid cells, astrocytes and neurons. We then classified each glioma cell into the subtype for which it had the highest score.

#### Classification of myeloid cell states

We used the CCA algorithm (Stuart et al., 2019) to classify our myeloid cell data into states defined in a previous scRNAseq study (Sankowski et al., 2019), using the 2000 most variable genes in the reference data set.

#### RT-*qPCR*

Taqman RT-qPCR (Thermo Fisher) inventoried human assays were used according to manufacturers’ instructions, probe IDs available on request.

### Quantification And Statistical Analysis

All statistical comparisons were undertaken using Graphpad Prism software v9.0.0. Summary statistics and specifics of statistical tests and their results are detailed in the figure legends. In the context of primary human samples, n refers to biological replicates corresponding to distinct primary patient/donor samples unless otherwise stated.

## Supplementary Material

Supplemental Items
