# Supplementary material for: Myeloid cell interferon secretion restricts Zika flavivirus infection of developing and malignant human neural progenitor cells
Source: Neuron. Author manuscript; Available in PMC 2024 Feb 1. (PMC7615581; doi:10.1016/j.neuron.2022.09.002)
Supplement: Supplemental Items [file EMS193590-supplement-Supplemental_Items.pdf]

Supplemental Items

Figure S1 Primary GBM and HDB slice culture models, Related to Figure 1

(A) ZIKV +strand smFISH in HDB - uninfected (MOCK) or infected with ZIKV and fixed at the times indicated. Hours or days post infection (h.p.i) or (d.p.i). Box shows area magnified in lower row.

(B) smFISH in HR GBM 042 MOCK and fixed at the equivalent of 72 h.p.i. Box shows area magnified in lower row.

(C) smFISH for ZIKV 7 d.p.i. in matched forebrain and hindbrain HDB slice cultures harvested at 8 weeks gestation. Insets show magnified region.

(D) IF Costaining for flavivirus envelope protein (ZIKVE) and SOX2 in HDB and GBM slice cultures at 72 h.p.i.

(E) smFISH of GBM slice culture at 7 d.p.i. ZIKV+ Strand is ZIKV genomic RNA, ZIKV- Strand is ZIKV replication intermediate.

(F) IF for flavivirus envelope protein (ZIKVE) and cleaved caspase 3 (Cl-Casp3) in HDB and GBM tissue (outlined in white) at 7 d.p.i. (ZIKV 1x10^7 PFU per well). Box shows area magnified in lower rows. Arrows point to Cl-Casp3 positive cells.

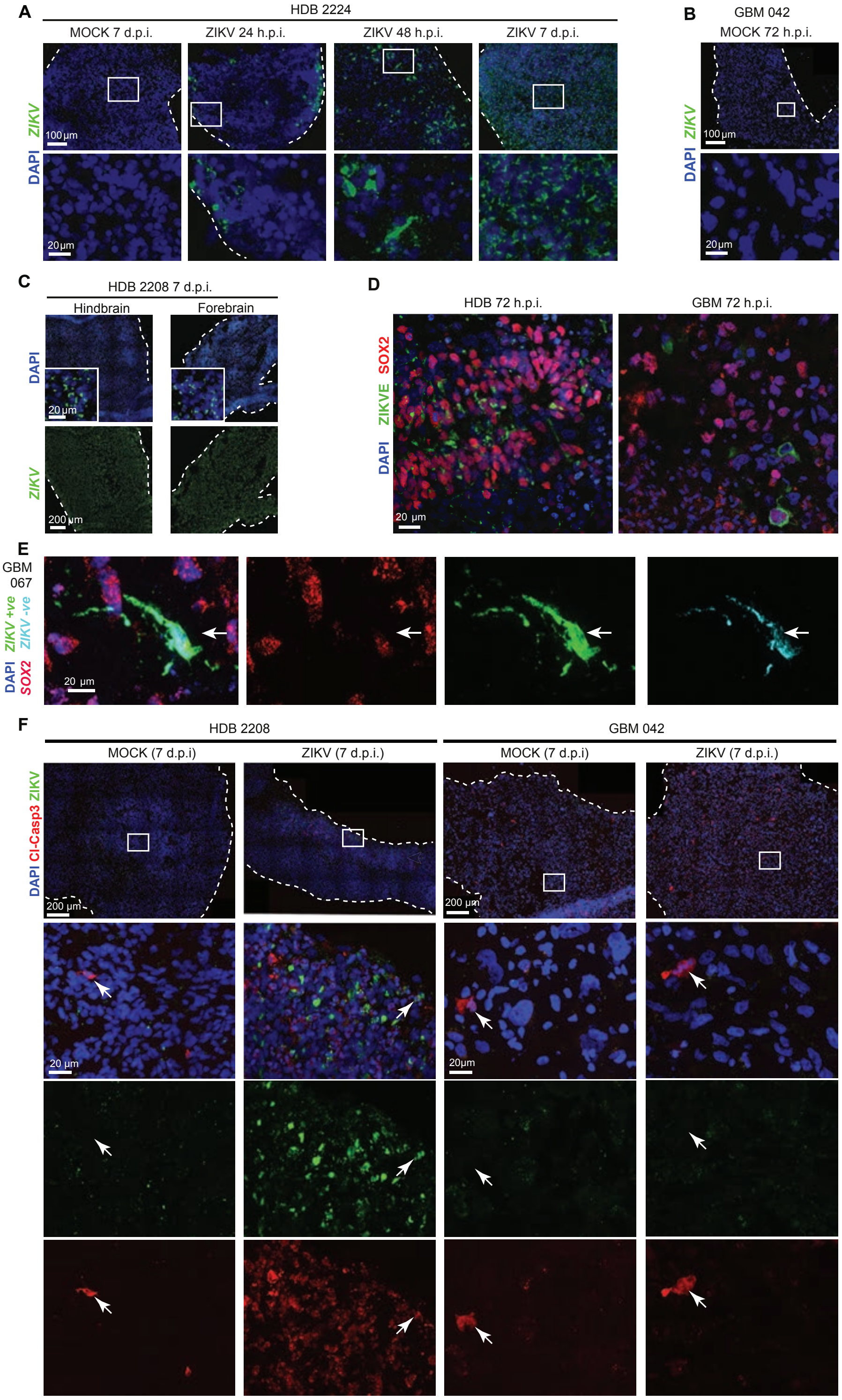
A
HDB 2224
MOCK 7 d.p.i.
ZIKV 24 h.p.i.
ZIKV 48 h.p.i.
ZIKV 7 d.p.i.
ZIKV
DAPI
100 μm
20 μm
B
GBM 042
MOCK 72 h.p.i.
ZIKV
DAPI
100 μm
20 μm
C
HDB 2208 7 d.p.i.
Hindbrain
Forebrain
DAPI
ZIKV
20 μm
200 μm
D
HDB 72 h.p.i.
GBM 72 h.p.i.
DAPI ZIKVE SOX2
20 μm
E
GBM 067
DAPI SOX2 ZIKV +ve ZIKV -ve
20 μm
F
HDB 2208
GBM 042
MOCK (7 d.p.i)
ZIKV (7 d.p.i.)
MOCK (7 d.p.i)
ZIKV (7 d.p.i.)
DAPI Cl-Casp3 ZIKV
200 μm
20 μm
200 μm
20μm

Figure S2 Primary GBM and HDB dissociated adherent culture model, Related to Figure 2

(A) RT-qPCR of ZIKV in low passage human developing forebrain and hindbrain lines HDB FB1 and HB1 (10 weeks); HDB FB12 and HB12 (12 weeks); H3K27M-bearing diffuse midline glioma lines DMG 007, DMG B169 and DMG B117; and GBM lines (E22, E25, E34). (*n=3-5*; ZIKV MOI 1, 48 h.p.i.; t-test HDB vs GBM ** p<0.01)

(B) Viable cells per well (biological triplicate) on infection of HDB FB1 and GBM E22 lines with ZIKV MOI 1 at time 0, compared to mock infected control (left panel) with representative live/dead imaging at time 72 h.p.i. (right panel).

(C) IF for selected lineage and cell cycle markers as indicated, in dissociated primary GBM and HDB cultures.

(D) IF to assess coexpression of astrocyte lineage and stem cell markers in the primary GBM malignant cell fraction. Arrows indicate cells where SOX2 colocalises with other stem cell markers.

(E) IF Costaining for flavivirus envelope protein (ZIKVE) and SOX2 in HDB and GBM dissociated cultures at 72 h.p.i.

(F) Proportion of MKI67+ cells in primary GBM tissue – independent clinical laboratory assessment.

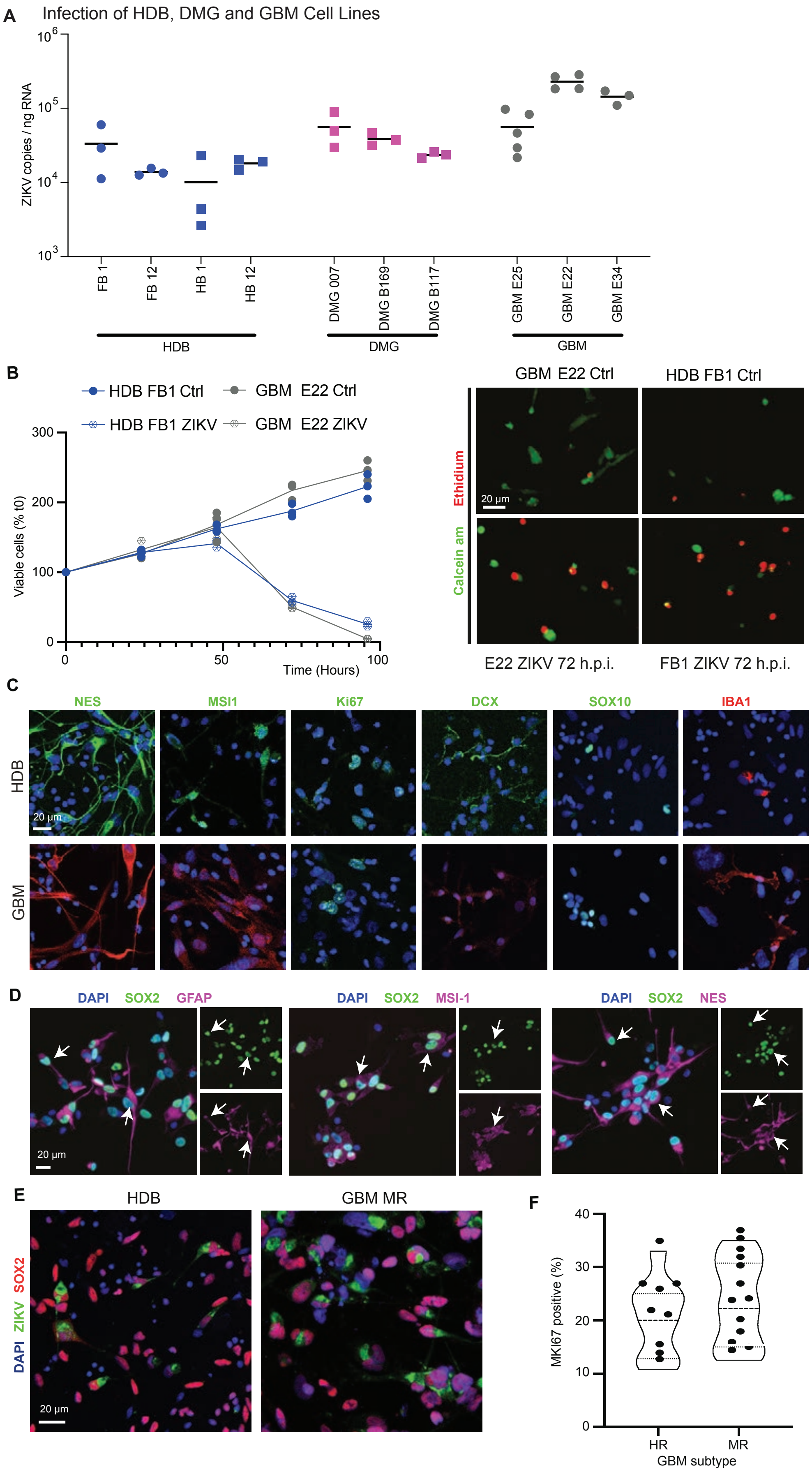
A
Infection of HDB, DMG and GBM Cell Lines
ZIKV copies / ng RNA
FB 1
FB 12
HB 1
HB 12
DMG 007
DMG B169
DMG B117
GBM E25
GBM E22
GBM E34
HDB
DMG
GBM
B
HDB FB1 Ctrl
GBM E22 Ctrl
HDB FB1 ZIKV
GBM E22 ZIKV
Viable cells (% t0)
Time (Hours)
GBM E22 Ctrl
HDB FB1 Ctrl
Ethidium
Calcein am
20 μm
E22 ZIKV 72 h.p.i.
FB1 ZIKV 72 h.p.i.
C
NES
MSI1
Ki67
DCX
SOX10
IBA1
HDB
GBM
20 μm
D
DAPI SOX2 GFAP
DAPI SOX2 MSI-1
DAPI SOX2 NES
20 μm
E
HDB
GBM MR
DAPI ZIKV SOX2
20 μm
F
MKI67 positive (%)
HR
MR
GBM subtype

Figure S3 ZIKV-mCh infection/ FACS sorting/ Bulk RNA-Sequencing, Related to Figure 3

(A) Percentage of total RNA-Seq reads aligning to the ZIKV genome in libraries derived from FACS-sorted ZIKV-mCh negative and positive cell fractions (n = 11 ZIKV-mCh+ and n = 11 paired ZIKV-mCh- libraries; box plots denote range with central bar corresponding to mean).

(B) Proportions of bulk primary cell population expressing ZIKV-mCh reporter 72 hpi (GBM) or 48 hpi (HDB) by FACS, GBM samples colour coded by TCGA subtype assigned where RNA-Seq data available.

(C) Expression of TCGA proneural, classical or mesenchymal signature genes by tumour sample.

(D) Expression of gene targets of interest in GBM (HR/MR/NOS) and HDB.

(E) Inferred immune cell content from bulk RNA-Seq libraries, using CIBERSORT (https://cibersort.stanford.edu/) LM22 gene signature matrix, collapsing inferred cell types into the categories shown.

(F) Proportion of cells IBA1 positive in representative primary GBM and HDB tissues (mean +/- s.e.m)

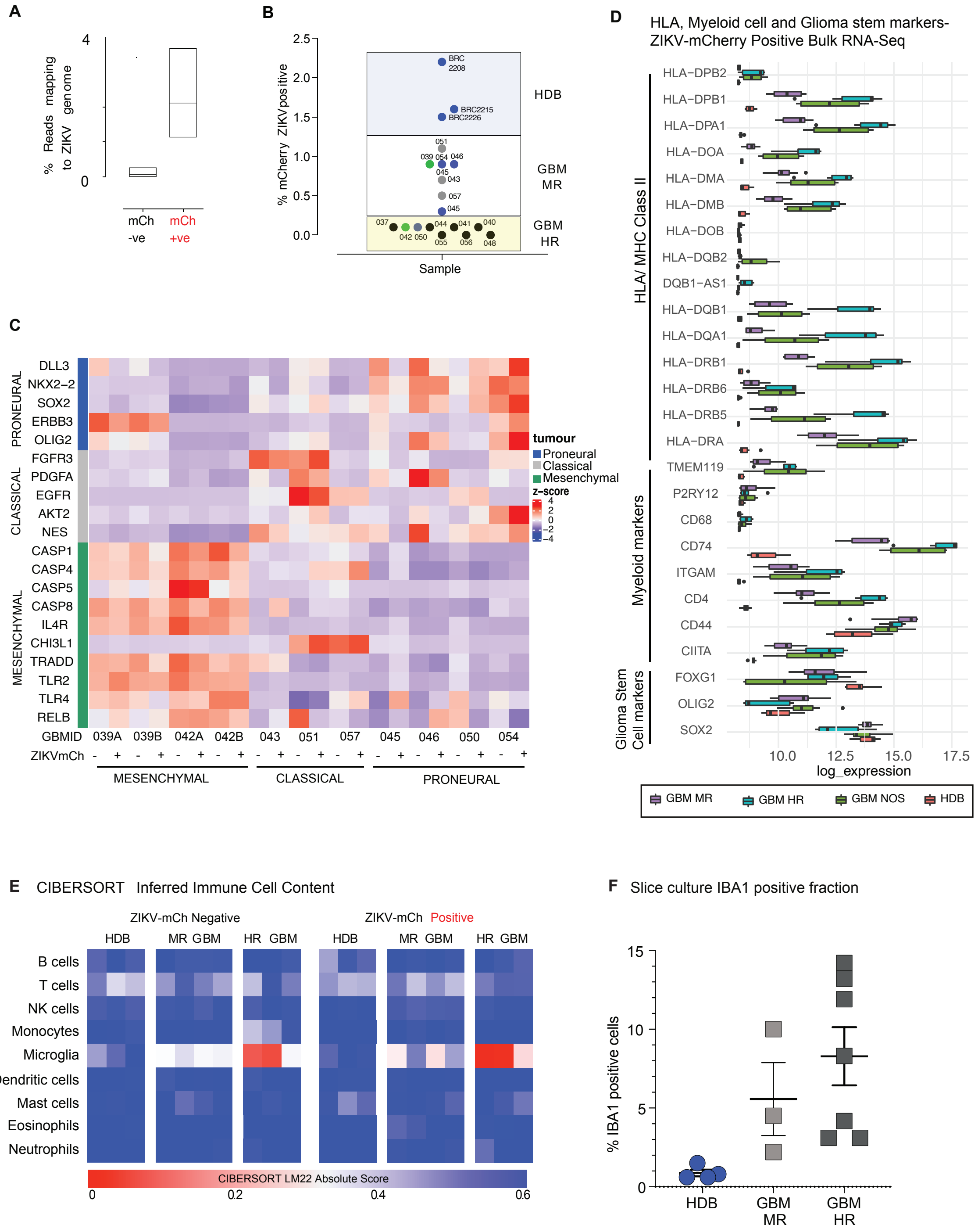
A
% Reads mapping to ZIKV genome
4
0
mCh -ve
mCh +ve
B
% mCherry ZIKV positive
2.5
2.0
1.5
1.0
0.5
0.0
BRC 2208
BRC2215
BRC2226
HDB
GBM MR
GBM HR
051
039 054 046
045
043
057
045
037
042 050
044
055
041
056
040
048
Sample
C
DLL3
NKX2−2
SOX2
ERBB3
OLIG2
FGFR3
PDGFA
EGFR
AKT2
NES
CASP1
CASP4
CASP5
CASP8
IL4R
CHI3L1
TRADD
TLR2
TLR4
RELB
PRONEURAL
CLASSICAL
MESENCHYMAL
tumour
Proneural
Classical
Mesenchymal
z−score
4
2
0
−2
−4
GBMID 039A 039B 042A 042B 043 051 057 045 046 050 054
ZIKVmCh - + - + - + - + - + - + - + - + - + - + - +
MESENCHYMAL
CLASSICAL
PRONEURAL
D
HLA, Myeloid cell and Glioma stem markers-
ZIKV-mCherry Positive Bulk RNA-Seq
HLA/ MHC Class II
HLA−DPB2
HLA−DPB1
HLA−DPA1
HLA−DOA
HLA−DMA
HLA−DMB
HLA−DOB
HLA−DQB2
DQB1−AS1
HLA−DQB1
HLA−DQA1
HLA−DRB1
HLA−DRB6
HLA−DRB5
HLA−DRA
Myeloid markers
TMEM119
P2RY12
CD68
CD74
ITGAM
CD4
CD44
CIITA
Glioma Stem Cell markers
FOXG1
OLIG2
SOX2
10.0
12.5
15.0
17.5
log_expression
GBM MR
GBM HR
GBM NOS
HDB
E
CIBERSORT Inferred Immune Cell Content
ZIKV-mCh Negative
ZIKV-mCh Positive
HDB
MR GBM
HR GBM
B cells
T cells
NK cells
Monocytes
Microglia
Dendritic cells
Mast cells
Eosinophils
Neutrophils
CIBERSORT LM22 Absolute Score
0
0.2
0.4
0.6
F
Slice culture IBA1 positive fraction
% IBA1 positive cells
15
10
5
0
HDB
GBM MR
GBM HR

Figure S4 ZIKV-mCh infection/ FACS sorting/ sc-RNA-Sequencing, Related to Figure 4

(A) glioma cell subtype identity scores (20) for all ZIKV-mCh+ glioma cells profiled (n=1392), arranged by overall subtype identity assigned.

(B) smFISH/IF for ZIKV and myeloid/glioma stem cell subtype markers in infected GBM tissue.

(C) Pearson Correlation coefficient between scRNA-Seq gene expression profile and LM22 immune cell gene expression signatures for all ZIKV-mCh+ immune cells.

(D) Proportions of each glioma cell subtype comprising the ZIKV-mCh+ glioma cell fraction isolated from MR and HR GBMs.

(E) ZIKV reads per ZIKV-mCh+ single cell, divided according to whether SOX2 was also detected (n = 1099; median 1172 reads per cell) or not detected (n = 627; median 591 reads per cell). **** $p<0.0001$ Mann Whitney U test 2-tail, red bars indicate median and interquartile range.

(F) Immunofluorescence for selected myeloid cell markers as indicated in primary GBM dissociated cultures.

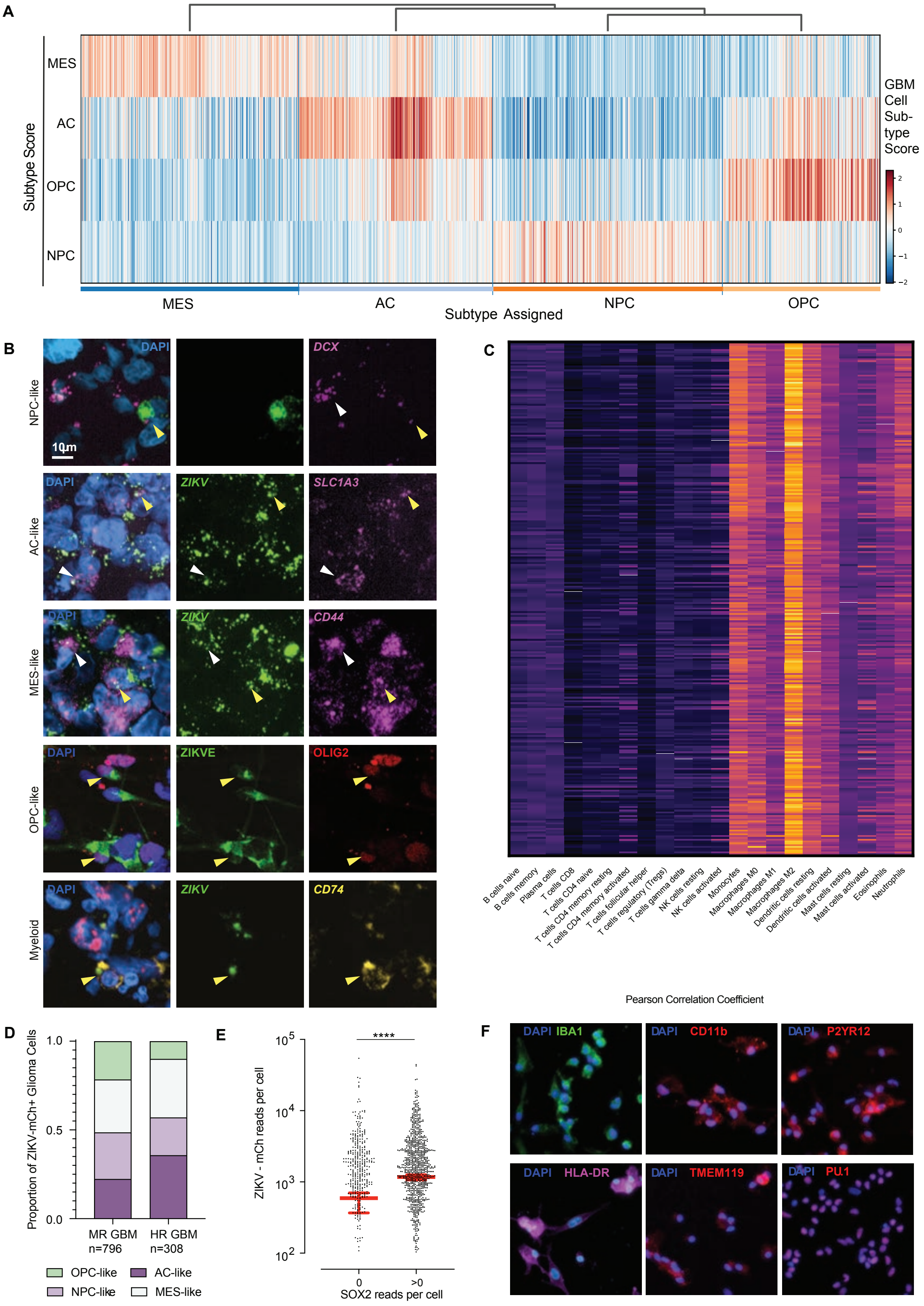
A
MES
AC
OPC
NPC
Subtype Score
GBM Cell Sub-type Score
2
1
0
−1
−2
MES
AC
NPC
OPC
Subtype Assigned
B
NPC-like
AC-like
MES-like
OPC-like
Myeloid
DAPI
DCX
10μm
ZIKV
SLC1A3
CD44
ZIKVE
OLIG2
CD74
C
B cells naive
B cells memory
Plasma cells
T cells CD8
T cells CD4 naive
T cells CD4 memory resting
T cells CD4 memory activated
T cells follicular helper
T cells regulatory (Tregs)
T cells gamma delta
NK cells resting
NK cells activated
Monocytes
Macrophages M0
Macrophages M1
Macrophages M2
Dendritic cells resting
Dendritic cells activated
Mast cells resting
Mast cells activated
Eosinophils
Neutrophils
Pearson Correlation Coefficient
D
Proportion of ZIKV-mCh+ Glioma Cells
1.0
0.5
0.0
MR GBM n=796
HR GBM n=308
OPC-like
AC-like
NPC-like
MES-like
E
ZIKV - mCh reads per cell
10^5
10^4
10^3
10^2
****
0
>0
SOX2 reads per cell
F
DAPI IBA1
DAPI CD11b
DAPI P2YR12
DAPI HLA-DR
DAPI TMEM119
DAPI PU.1

Figure S5 GBM CD11b MACS Sorting and Conditioned Media Assays, Related to Figures 5, 6 and 7

(A) Western for IBA1 and SOX2 expression in parent GBM and sorted GBM11b- and GBM11b+ fractions.

(B) IF for flavivirus envelope protein in GBM E34 cultures and in 2:1 coculture with GBM11b+ or GBM11b- fractions as per main figure.

(C) IF for flavivirus envelope and IBA1 in ZIKV-infected pure HDB cultures (left) and HDB cultures supplemented with GBM myeloid cell (right) (MOI 1 48 h.p.i.)

(D) IF for IBA1 in HDB cells after MACS for CD11b expression.

(E) Top gene ontology enrichment terms for 154 genes differentially expressed in 3 glioma cell and NSC lines between LineCM and 11b+CM conditions (g:profiler: Reactome pathway database https://doi.org/10.1093/database/baz123).

(F) Expression of selected interferon stimulated genes (ISGs) and glioma cell transcription master regulators in GBM E22 and GBM E34 cells in LineCM and 11b+CM conditions.

(G) ZIKV RT-qPCR of GBM E22 cultures 48 h.p.i. treated with DMSO control, MIP-1α (20 ng/ml), MIP-1β (20 ng/ml) as indicated.

(H) ZIKVE IF in GBM E22 cells 48 h.p.i., with or without 24 h pre-treatment with recombinant IFNβ (100 pg/ml) +/- ruxolitinib 1000 nM.

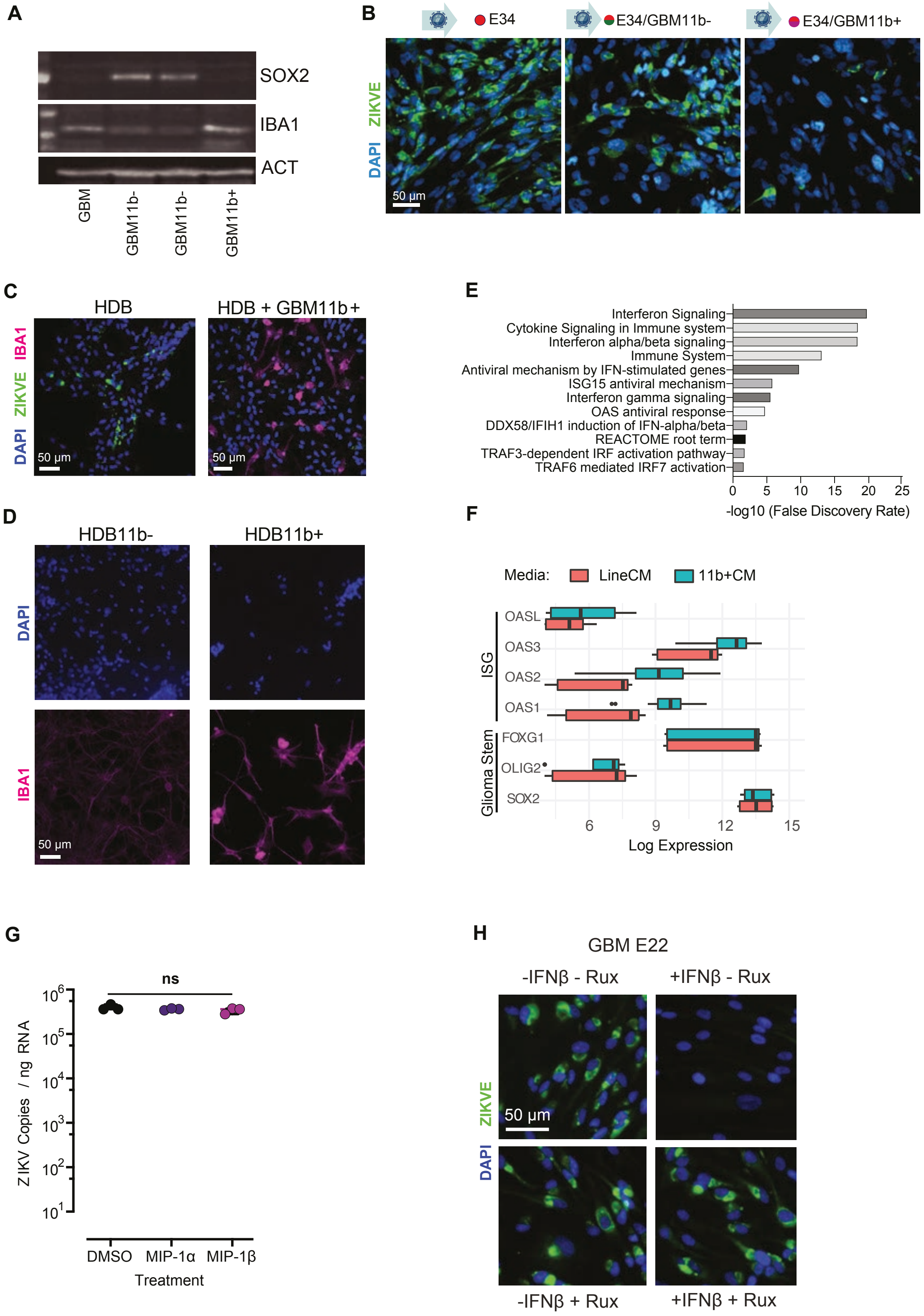
A
SOX2
IBA1
ACT
GBM
GBM11b-
GBM11b-
GBM11b+
B
E34
E34/GBM11b-
E34/GBM11b+
ZIKVE
DAPI
50 µm
C
HDB
HDB + GBM11b+
IBA1
ZIKVE
DAPI
50 µm
50 µm
D
HDB11b-
HDB11b+
DAPI
IBA1
50 µm
E
Interferon Signaling
Cytokine Signaling in Immune system
Interferon alpha/beta signaling
Immune System
Antiviral mechanism by IFN-stimulated genes
ISG15 antiviral mechanism
Interferon gamma signaling
OAS antiviral response
DDX58/IFIH1 induction of IFN-alpha/beta
REACTOME root term
TRAF3-dependent IRF activation pathway
TRAF6 mediated IRF7 activation
0 5 10 15 20 25
-log10 (False Discovery Rate)
F
Media: LineCM 11b+CM
ISG
OASL
OAS3
OAS2
OAS1
Glioma Stem
FOXG1
OLIG2
SOX2
6 9 12 15
Log Expression
G
ns
ZIKV Copies / ng RNA
10^6
10^5
10^4
10^3
10^2
10^1
DMSO
MIP-1α
MIP-1β
Treatment
H
GBM E22
-IFNβ - Rux
+IFNβ - Rux
ZIKVE
DAPI
50 µm
-IFNβ + Rux
+IFNβ + Rux

Supplemental Table S1: Abbreviations, Related to all text and figures

Cl-Casp3	Cleaved caspase3
CM	Conditioned media
d.p.i.	Days post infection
FACS	Fluorescence activated cell sorting
GBM	Glioblastoma
GBM11b+	GBM CD11b+ sorted cell fraction
h.p.i.	Hours post infection
HDB	Human developing brain
HLA	*Human leucocyte antigen*
HR	Highly refractory
IF	Immunofluorescence
IFNβ	Interferon beta
ISG	Interferon-stimulated genes
JAK	Janus kinase
MACS	Magnetic activated cell sorting
mCh	mCherry
MOI	Multiplicity of infection
MR	Moderately refractory
MSD	Meso scale discovery
RT-qPCR	Real time quantitative polymerase chain reaction
Rux	Ruxolitinib
scRNA seq	Single cell RNA sequencing
smFISH	Single molecule fluorescent *in situ* hybridization
TCGA	The cancer genome atlas
TME	Tumour microenvironment
ZIKV	Zika virus
ZIKVE	Flavivirus envelope protein
ZIKV-mCh	Zika Virus-mCherry

Conditioned media abbreviations:

11b+CM	Conditioned media from CD11b+ cell fraction
11b-CM	Conditioned media from CD11b- cell fraction
Pi:c_11b+CM	Conditioned media from CD11b+ cell fraction pretreated with poly(I:C).
LineCM	Conditioned media harvested from GBM or FNS cell lines

Supplemental Table S2: Primary GBM Patient demographics and routine histopathological analysis, Related to STAR Methods

Study ID	Age	Sex	Pathology	IDH mutation	MGMT Hypermethylation	Slice Culture	Dissociated Cells
30	62	M	GBM IV	-	-		x
31	58	M	GBM IV	-	-		x
37	56	F	GBM IV	-	+	X	x
39	73	F	Gliosarcoma IV	-	-		x
41	51	F	GBM IV	-	+		x
42	77	M	GBM IV	-	-	x	x
43	51	M	GBM IV	-	-		x
45	57	F	GBM IV	-	-		x
46	78	M	GBM IV	-	-		x
47	71	M	GBM IV	-	-	x	x
48	49	F	GBM IV	-	-		x
50	57	M	GBM IV	-	-		x
51	59	M	GBM IV	-	-		x
53	60	M	GBM IV	-	-		x
54	54	M	GBM IV	-	-		x
55	58	M	GBM IV	-	-		x
56	68	M	GBM IV	-	-		x
57	69	F	GBM IV	-	-		x
58	56	F	GBM IV	-	-		x
59	66	F	GBM IV	-	+		x
60	56	M	GBM IV	-	+		x
62	67	M	Gliosarcoma IV	-	+		x
63	65	M	GBM IV	-	-		x
66	76	M	GBM IV	-	-		x
67	46	M	GBM IV	-	-	x	x
68	54	M	GBM IV	-	+		x

69	59	F	GBM IV	-	-		x
70	75	M	GBM IV	-	-		x
71	38	M	GBM IV	-	-		x
72	63	M	GBM IV	-	-		x
73	61	M	GBM IV	-	+		x
74	58	M	GBM IV	-	-		x
75	70	M	Gliosarcoma IV	-	-		x

Supplemental Table S3: Primary HDB sample characteristics, Related to STAR Methods

BRC Study ID	Age	Sex	Brain Region	Slice Culture	Dissociated Cells
2196	10w	Not determined	FB		x
2208	11w	Not determined	FB and HB	x	x
2223	8w6	Not determined	FB		x
2215	9w3	Not determined	FB		x
2224	9w2	Not determined	FB	x	
2225	11w4	Not determined	FB and HB	x	x
2226	9w5	Not determined	FB		x
2233	8w6	Not determined	FB and HB		x
2229	8w1	Not determined	FB		x
2251	9w1	Not determined	FB		x
2279	9w1	Not determined	FB		x
2285	11w1	Not determined	FB		x
2293	12w	Not determined	FB		x
2413	7w6	Not determined	FB	x	

Supplemental Table S4: Cell line names, origins and source, Related to STAR Methods

Line name	Origin	Source	Synonyms/ Reference
HDB-FB1	Human Developing Forebrain	Steve Pollard, Edinburgh (gcgr.org.uk)	FNS1 - Forebrain
HDB-FB12	Human Developing Forebrain	Steve Pollard, Edinburgh (gcgr.org.uk)	FNS12 – Region 1
HDB-HB1	Human Developing Hindbrain	Steve Pollard, Edinburgh (gcgr.org.uk)	FNS1 - Hindbrain
HDB-HB12	Human Developing Hindbrain	Steve Pollard, Edinburgh (gcgr.org.uk)	FNS12 – Region 6
DMG 007	Diffuse Midline Glioma	Angel Carcaboso, Barcelona	HSJD-DIPG-007
DMG B117	Diffuse Midline Glioma	Chris Jones, ICR London	ICR-B117
DMG B169	Diffuse Midline Glioma	Chris Jones, ICR London	ICR-B169
GBM-E22	Glioblastoma	Steve Pollard, Edinburgh (gcgr.org.uk)	E22
GBM-E25	Glioblastoma	Steve Pollard, Edinburgh (gcgr.org.uk)	E25
GBM-E34	Glioblastoma	Steve Pollard, Edinburgh (gcgr.org.uk)	4

Supplemental Table S5: Antibodies used in this study, Related to STAR Methods

**Primary Antibody**	**Supplier**	**Catalogue number**	**Species**	**Application**	**Dilution**
IBA-1 (Wako)	Alpha Laboratories	019-19741	Rabbit	IF	1:500
				Western	1:1000
Human SOX2	R & D Systems	AF2018	Goat	IF	1:500
				Western	1:1000
Flavivirus Group Antigen, clone D1-4G2-4-15	Millipore Sigma	MAB10216	Mouse	IF	1:300
Flavivirus Group Antigen, clone D1-4G2-4-15	Absolute Antibody	Ab00230-23.0	Rabbit	IF	1:200
Human Nestin MAb (Clone 196908)		MAB1259	Mouse	IF	1:100
OLIG2	Millipore Sigma	AB9610	Rabbit	IF	1:200
GFAP Monoclonal Antibody (2.2B10)	ThermoFisher Scientific	13-0300	Rat	IF	1:500
Tuj1 Neuron-specific beta-III tubulin	Millipore Sigma	MAB1195?	Mouse	IF	1:1000
Ki67 (SP6)	Abcam	ab16667	Rabbit	IF	1:500
Recombinant Anti-Ki67 antibody [37C7-12]	Abcam	ab245113	Mouse	IF	1:300
MSI-1	MBL	D270-3	Rat	IF	1:500
Cleaved Caspase 3 (Asp175) Antibody	Cell Signalling	9661S	Rabbit	IF	1:300
Pax6	DSHB		Mouse	IF	1:200
CUX2	Abcam	ab216588	Rabbit	IF	1:500
SOX10	R & D Systems	AF2864	Goat	IF	1:500
STAT-1	Cell Signalling	9176S	Mouse	Western	1:1000
Phospho-Stat1 (Tyr701) (D4A7)	Cell Signalling	7649	Rabbit	Western	1:500
Anti-β-Actin	Sigma Aldrich	A1978	Mouse	Western	1:5000
Secondary Antibody	Supplier	Catalogue number	Species	Application	Dilution
Donkey anti-Goat IgG (H+L) Cross-Adsorbed	Life Technologies	A-11055	Donkey	IF	1:500

Secondary Antibody, Alexa Fluor 488					
Donkey anti-Mouse IgG (H+L) Highly Cross-Adsorbed Secondary Antibody, Alexa Fluor 594	Life Technologies	A-21203	Donkey	IF	1:500
Donkey anti-Mouse IgG (H+L) Highly Cross-Adsorbed Secondary Antibody, Alexa Fluor 488	Life Technologies	A-21202	Donkey	IF	1:500
Donkey anti-Rabbit IgG (H+L) Highly Cross-Adsorbed Secondary Antibody, Alexa Fluor 594	Life Technologies	A-21207	Donkey	IF	1:500
Donkey anti-Mouse IgG (H+L) Highly Cross-Adsorbed Secondary Antibody, Alexa Fluor 647	Life Technologies	A-31571	Donkey	IF	1:500
Donkey anti-Rabbit IgG (H+L) Highly Cross-Adsorbed Secondary Antibody, Alexa Fluor 647	Life Technologies	A-31573	Donkey	IF	1:500
IRDye 680RD Goat Anti-Mouse IgG (H+L), 0.1 mg	LI-COR Biosciences	925-68070	Goat	Western	1:5000
IRDye 680RD Goat Anti-Rabbit IgG (H+L), 0.1 mg	LI-COR Biosciences	925-68071	Goat	Western	1:5000
IRDye 800CW Goat Anti-Rabbit IgG (H+L), 0.1 mg	LI-COR Biosciences	925-32211	Goat	Western	1:5000
IRDye 800CW Goat Anti-Mouse IgG (H+L), 0.1 mg	LI-COR Biosciences	925-32210	Goat	Western	1:5000
IRDye 680RD Donkey Anti-Goat IgG (H+L), 0.1 mg	LI-COR Biosciences	925-68074	Donkey	Western	1:5000

Table S6: smFISH ACD RNA-Scope Probes, Related to STAR Methods

symbol	Species	Channel	Probe.Name	gene.name	Catalog.ACD	accession	probe_region	description
ZIKV	Zika virus	C1	V-ZIKV	Zika Virus	467778	N/A	219 - 5443	80 pairs covering Consensus sequence (ZIKV)
ZIKV	Zika virus	C4	V-ZIKV	Zika Virus	467778-C4	N/A	219 - 5443	80 pairs covering Consensus sequence (ZIKV)
ZIKV	Zika virus	C2	V-ZIKA-pp-O3-sense-C2	Zika Virus	467911-C2	KJ776791.1	5756 - 7866	Custom probe covering 40 pairs covering Consensus sequence (ZIKV)
SOX2	Hs	C2	Hs-SOX2-C2	SOX2 (SRY-BOX 2)	400878	NM_003106.3	730 - 2242	Homo sapiens SRY (sex determining region Y)-box 2 (SOX2), mRNA
RUNX1	Hs	C3	Hs-RUNX1-C3	Runt-related transcription factor 1	419908-C3	NM_001122607.1	284 - 1430	Homo sapiens runt-related transcription factor 1 (RUNX1) transcript variant 3 mRNA
RUNX1	Hs	C2	Hs-RUNX1-C2	Runt-related transcription factor 1	419908-C2	NM_001122607.1	284 - 1430	Homo sapiens runt-related transcription factor 1 (RUNX1) transcript variant 3 mRNA
OLIG2	Hs	C2	Hs-OLIG2-C2	Oligodendrocyte lineage transcription factor 2	424198-C2	NM_005806.3	929 - 2502	Homo sapiens oligodendrocyte lineage transcription factor 2 (OLIG2) mRNA
DCX	Hs	C2	HS-DCX-C2	Doublecortin	489558-C2	NM_000555.3	181 - 1381	Homo sapiens doublecortin (DCX) transcript variant 1 mRNA
CD74	Hs	C1	HS-CD74	Cluster of Differentiation 74	477528	NM_004355.3	49 - 1167	Homo sapiens CD74 molecule (CD74) transcript variant 2 mRNA
Cd44	Hs	C3	HS-CD44	CD44	311278-C3	NM_000610	157 - 1435	Homo sapiens CD44 molecule (Indian blood group) (CD44) transcript variant 1 mRNA
SLC1-A3	Hs	C1	HS-SLC1A3	Solute carrier family 1 (glial high affinity glutamate transporter) member 3	461088	NM_004172.4	2 - 1226	Homo sapiens solute carrier family 1 (glial high affinity glutamate transporter) member 3 (SLC1A3) transcript variant GLAST mRNA
PDGFRA	Hs	C1	HS-PDGFRA	Platelet-derived growth factor receptor, alpha polypeptide	604488	NM_006206.4	844 - 1774	Homo sapiens platelet-derived growth factor receptor, alpha polypeptide (PDGFRA), mRNA
Negative	N/A		RNAscope 4plex negative control	RNAscope 4plex negative control probe DapB	321838	N/A		RNAscope® 4-plex LS Multiplex Negative control probe DapB (of Bacillus subtilis strain) for RNAscope® LS 2.5 Multiplex Fluorescent Assay.

Table S7: Operetta imaging, Related to STAR Methods

Channel	Fluorophore	Excitation LED (nm)	Excitation LED	Excitation	Dichroic	Emission Filter (nm)	EM filter part info**	Exposure Time (ms)
			**Power (%)**	**Filter (nm)**	**LP (nm)***			
**DAPI**	DAPI	365	40	355-385	425	430-500	PE 430-500	60
**smFISH 1**	Atto 425	450	80	435-460	465	473-491	SR 482/18	120
**smFISH 2**	Opal 520	505	80	490-515	525	532-552	SR 542/20	120
**smFISH 3**	Opal 570	550	80	530-560	565	585-610	SR 598/25	120
**smFISH 4**	Opal 650	630	80	615-645	650	654-672	SR 663/18	120
*Dichroic longpass mirrors		**Emission filters purchased from Perkin Elmer (PE) or Semrock (SR)						
**Settings for the 40x scan for smFISH**								
**Channel**	**Fluoro-phore**	**Excitation LED (nm)**	**Excitation LED Power (%)**	**Excitation Filter (nm)**	**Dichroic LP (nm)***	**Emission Filter (nm)**	**EM filter part info****	**Exposure Time (ms)**
**DAPI**	DAPI	365	30	355-385	425	430-500	PE 430-500	40
**Alexa 488**	Alexa 488	450	50	460-490	525	500-550		40
**Alexa 594**	Alexa 594	505	50	530-560	565	570-650		40
**Alexa 647**	Alexa 647	550	50	615-645	650	655-672	SR 663/18	40
Settings for the 20x lens non-confocal scan for [illegible]								
